# How can an agent-based model explore the impact of interventions on children's physical activity in an urban environment?

**DOI:** 10.1016/j.healthplace.2021.102688

**Published:** 2021-11

**Authors:** Jonatan Almagor, Anne Martin, Paul McCrorie, Rich Mitchell

**Affiliations:** MRC/CSO Social and Public Health Sciences Unit, University of Glasgow, Berkeley Square, 99 Berkeley Street, G3 7HR, Glasgow, Scotland, UK

**Keywords:** Agent-based model, Physical activity, Children, Urban environment, Complex systems

## Abstract

Insufficient physical activity (PA) among most children and adolescents is a global problem that is undermining the realisation of numerous developmental and health benefits. The aim of this study was to explore the potential impact of interventions on PA by using an agent-based model (ABM) simulating children's daily activities in an urban environment. Three domains for interventions were explored: outdoor play, school physical education and active travel. Simulated interventions increased children's average daily moderate-to-vigorous PA by 2–13 min and reduced the percentage of children not meeting PA guidelines, from 34% to 10%–29%, depending on the intervention. Promotion of active travel and outdoor play benefited more those in a higher socio-economic position. Agents' interactions suggested that: encouraging activity in diverse groups will reduce percentage of the least active in the population; and initiating outdoor events in neighbourhoods can generate an enhancing effect on children's engagement in PA. The ABM provided measurable outcomes for interventions that are difficult to estimate using reductionist methods. We suggest that ABMs should be used more commonly to explore the complexity of the social-environmental PA system.

## Highlights

• We developed an agent-based model simulating children's daily physical activity.
• Agents perform typical daily activities in a virtual urban model of Glasgow city.
• Simulations explore impact of interventions on outdoor play, school, active travel.
• Promotion of active travel had a differential impact across socio-economic position.
• Outdoor play intervention produced a non-linear increase in physical activity.

## 1 Background

Physical activity (PA) is associated with numerous developmental and health benefits in children and adolescents (Janssen and LeBlanc, 2010; Poitras et al., 2016; Booth et al., 2011, 2017). According to the World Health Organization (WHO), children should accumulate at least 60 min of moderate-to-vigorous-intensity PA (MVPA) daily. However, WHO estimates suggest that only 19% of children aged 11–17 years are sufficiently physically active (WHO, 2010), and this is corroborated by surveys from various countries: 25% in USA (CDC, 2018), 18% in England (NHS, 2019), 30% in China (Fan and Cao, 2017), and 11% in Scotland (McCrorie et al., 2018).

In general, the more time children spend being physically active, the greater the health benefits. Gains are especially significant for those currently doing the lowest levels of activity, as the improvements in health per additional minute of physical activity will be proportionately greater (Foster et al., 2019). Therefore, identifying potential domains in children's daily schedule where the ‘dose’ of PA can be manipulated using different types of interventions, which both increase PA levels overall and specifically affect those least active, is a policy priority.

Studies using accelerometers in combination with location tracking devices demonstrate that PA intensity is associated with the location of an activity. These studies have found that some environments promote or enhance children's PA (Matisziw et al., 2016; Klinker et al., 2014; Coombes et al., 2013; Prince et al., 2019). For example, higher proportions of the time spent in MVPA were identified when children were outdoors in parks, gardens, open spaces, playing fields and schoolyards, as opposed to lower proportions when children were indoors at home or during school lessons. Studies also have shown that children's daily activities are mostly confined to the area in close proximity to their home, school and the paths that connects them (Remmers et al., 2019). This evidence has led to the suggestion that interventions focused on increasing use of and/or the availability and accessibility of outdoor environments for children would be effective in increasing their PA levels (Mölenberg et al., 2019; Stone and Faulkner, 2014).

However, we know that PA is the outcome of many interacting determinants operating at different levels: individual, social, environmental and policy. These are all part of one integrated social-environmental system of which environment or location is just one component (Sallis et al., 2006; Rutter et al., 2019). PA at the population level emerges from the behaviours and interactions of children, who respond to and influence one another while being affected by additional factors, including their physical living environment and social conditions that may reinforce or constrain their behaviour. Although the complexity of the PA system is acknowledged, typically the science undertaken to understand PA, and to develop or evaluate interventions, tends to be reductionist and focussed on one or two system components.

In this study, we formalise our understanding of the multiple interacting factors that form children's PA system by means of an agent-based model (ABM). We use the ABM to simulate the emergence of MVPA levels of children residing in an urban environment. We explore the influence of the contributing factors and evaluate the potential impact of several interventions in children's daily activity on MVPA levels.

ABMs are computational models that simulate complex social systems by representing agents that interact with one another and with the environment in which they live according to predefined rules. The micro-level behaviours of agents generate the dynamics of the system from the bottom-up and lead to the emergence of macro-level patterns observed at the population level. ABMs examine complex processes involving multiple dynamic interactions among individuals, and between individuals and their environments over time and space.

Initially ABMs were used in public health to simulate the spread of infectious disease (Perez and Dragicevic, 2009; Merler et al., 2015). Subsequently, ABMs have been developed for non-communicable disease and health behaviours including obesity (Giabbanelli et al., 2021), smoking (Luke et al., 2017), alcohol consumption (Gorman et al., 2006) and unhealthy food consumption (Auchincloss et al., 2011). By including interactions between agents, ABMs can account for the effect of social influence on establishment and adaptation of health behaviours in a population, while interactions with the physical environment can account for the differential exposures of individuals residing in different geographical locations. A key feature in ABMs is the representation of heterogeneity among agents’ characteristics and behaviours, which better reflects the diversity of a population and its impact on health.

ABMs developed for PA have been particularly used for the exploration of active travel, where geographical location and distance are essential components of an agent's decision. For example, Yang et al. (2011, 2012, 2015) developed a series of ABMs to explore adults' active travel. Using an abstract urban environment, including a population residing in neighbourhoods segregated by socio-economic class, the researchers tested how changes in safety, attitudes towards walking, land use distribution and transportation cost influenced socio-economic inequalities in active travel. Yang et al. (2014) used an ABM to evaluate the promotion of active travel to school using a walking “school bus” strategy. In this model, agents decide whether to join the walking “school bus”, walk on their own or be driven to school. Their decisions are based on multiple criteria, including distance, traffic safety, walking speed and waiting times. The model evaluated pupils' active travel under different scenarios.

ABMs also simulated the emergence and evolution of population patterns of leisure time PA among adults (Garcia et al., 2017) and explored how interventions can reduce inequalities in participation (Blok et al., 2018). The capability of ABMs to explicitly represent social networks and simulate dynamic interactions has also been employed to simulate peer influence on PA (Zhang et al., 2015; Fernandes de Mello Araújo et al., 2018). In these studies, a friendship network is represented and the ABM simulates the dynamic process of PA modification across the network following an intervention that increases PA levels of a subset of selected agents.

Implementing interventions aimed at increasing PA levels overall and influencing those least active is a policy priority. However, evaluating interventions is costly and difficult on a large scale and data on their impact is limited. The aim of this study was to explore the potential impact of interventions on PA by using a computational model that considers the complexity of the social-environmental system. In this study we developed an ABM that simulates typical daily activities of children that contribute to accumulation of MVPA. We then used the model to simulate various interventions in the daily activity schedule of agents to evaluate the potential impact on MVPA levels in the population and among socio-economic groups. Using the model, we explored how factors related to agents’ characteristics, their interactions and the environment to which they were exposed impact on PA.

## 2 Methods

### 2.1 Model overview

The model simulates the daily weekday schedule of 9-11-year-olds and the PA they accumulate throughout the day. Agents are embedded in an urban environment represented by geographical information data such as buildings, roads and land-use based on the city of Glasgow, Scotland. The model includes a heterogenous population of agents characterised by: gender, socio-economic position (SEP), car availability and participation in formal sport activities (FSA), based on Glasgow's census statistics and sport participation survey. In addition, agents vary in preferences to engage in outdoor play and in their tendency to be active.

The agents follow a daily schedule that includes attending school, meeting friends, outdoor free play, participation in sport clubs and shopping. The agents make repeated decisions regarding the mode of travel, participation in activities and selection of sites, while considering the social and environmental conditions together with their own preferences. In addition, agents’ PA and participation in certain activities are influenced by the behaviour of their peers. As the agents engage in activities and travel, they accumulate PA, measured as minutes of MVPA.

The model runs over a period of 30 days during which the distribution of MVPA levels in the population and across the city emerges. The model is implemented using the GAMA-Platform simulation environment (GAMA) and can be freely downloaded from (Almagor, 2021). In what follows, we describe the theoretical rationale behind the model and present the formal implementation. For further details see ODD + D protocol (Supplementary material.1).

### 2.2 Theoretical and empirical background

The social-ecological framework provides a theoretical background for the ABM. We assume the PA of an individual is influenced by interrelated factors on multiple levels: **individual, social environment, urban environment and policy** (Sallis et al., 2006). Factors related to these multiple levels are integrated within the model, affecting the agents’ decision-making, their activity schedule and behaviour. Collectively, this simulates a complex system from which individual PA and, subsequently, population levels of PA emerge. The influence of each level is theorised and then implemented by drawing on empirical evidence and the literature. Below, we set out the ideas and relationships that are simulated. Then, we show in more detail how these are implemented in the model.

At the **individual level** agents are characterised by SEP and PA-relevant attributes that reflect economic constrains associated with SEP; these include availability of cars in the household and participation in formal sport activities (e.g. sports clubs). Car availability affects the travel mode selection (car vs. walking) and participation in formal sport activities affects the activity schedule of the agent. Both, in turn, affect levels of PA.

As for the intensity level of PA, it differs by agent's gender, as we observed in empirical data (Fig. 3). Moreover, psychological theories assert that variation in PA behaviour is the outcome of a complex interplay between psychological variables (Hagger et al., 2001; Rhodes et al., 2019). Although our model does not explicitly represent the levels of psychological variables, we do include an abstract variable representing a ‘tendency’ to be active that introduces variation between agents: given the same activity, depending on their ‘tendency’, some agents are more likely to accumulate MVPA while others are less so. In addition, we assume that agents differ in their willingness to engage in outdoor activity. This could be because of differing preferences (Cleland et al., 2010) and/or parental influence (Remmers et al., 2014).

The influence of the **social environment** is based on findings suggesting that children adapt thier PA behaviour in accordance with the behaviour of friends (Macdonald-Wallis et al., 2012; Maturo and Cunningham, 2013; Stearns et al., 2019). Moreover, children may be more attracted to participate in activities when and where other children are present (Floyd et al., 2011; Pedroni et al., 2019). To implement these influences, social ties are created between agents to represent a friendship network. Agents adapt their tendencies and preferences to those of their friends when performing activities together. In addition, agents sense the presence of other agents in their vicinity and are more likely to participate in outdoor activity when more agents are present.

Evidence suggests that adverse social conditions in the neighbourhood may lead to concerns about safety and discourage activity outdoors (Rees-Punia et al., 2018; Marquet et al., 2019). To reflect this, an agent's likelihood to participate in an outdoor activity in the neighbourhood is affected by deprivation level. Neighbourhood deprivation levels in the model are based on the Scottish Index of Multiple Deprivation (SIMD) (Scottish Government, 2020). The index is a combined measure of the extent to which an area is deprived across seven domains: income, employment, education, health, access to services, crime and housing.

The urban **environment** provides places to engage in PA behaviour. Land use types differ in the activities they afford and facilitate, and are associated with varying intensity levels of PA (Prince et al., 2019). In the model, we assume that different types of land use and sites can be characterised by the PA intensity that children perform when spending time there. To represent the impact of land use on intensity level of PA, each type of land use in the model is assigned a parameter that controls the intensity level of PA performed by the agents when they are located there. Land use parameters that control PA were derived from an analysis of empirical data of children's MVPA accumulation at different land use and facilities (section: Physical activity).

The configuration of the living environment may also affect accessibility to sites for engagement in outdoor activity. Distance is a key consideration for selecting a site for recreational activities (Koppen et al., 2014; Dunton et al., 2014; Van Hecke et al., 2016). In the model, distance to a site as well as the social influence (presence of others at the location) and the size of the site are considered by the agents when they select a location for outdoor activity.

Distance and street walkability are central determinants of children's choice for mode of travel (Oliver et al., 2015; Williams et al., 2018; Macdonald et al., 2019), thus affecting PA accumulated by active travel. In the model, roads are explicitly represented allowing agents to evaluate walkability and distance before every journey to decide on the preferred mode of travel. Empirical data from children's travel diaries are used to estimate a probability function used by the agents to decide on the mode of travel (car vs. walk).

In this simulation, **policy** is represented as different intervention scenarios. In the scenarios we manipulate parameters that control the engagement of the agents in specific activities during the week and observe the impact on PA levels in the population. More specifically we test three potential intervention domains in the children's day: school, outdoor play in the neighbourhood and active travel.

### 2.3 Modelling the urban environment

**Fig. 1:**
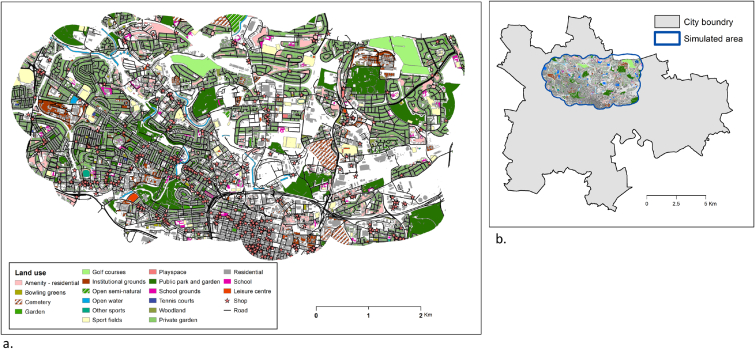
The urban environment. (a) Georefrenced vector layers of land use, buildings and road network form the urban environment. (b) The environment covers 34 km^2^ of the city of Glasgow.

An area in the city of Glasgow consisting of 120 Data Zones units, covering 34 km2, is used as the setting for the simulation (Fig. 1). Data Zones are small geographical areas used in the collection and reporting of administrative and census data in Scotland. The data zones include demographic statistics and the level of deprivation (SIMD) of the area, divided into 5 levels based on quintiles. Georeferenced data layers of land use, houses, schools, shops and the road network represent the urban environment (sources: Scottish Greenspace Map, Ordnance Survey MasterMap and points of interest) (Fig. 1a). This landscape provides the spatial context in which the daily activities of the agents take place. The road network is used by agents for travel and their activities are affected by geographical constrains such as distance, availability of facilities and land use around their home and school.

### 2.4 Agents

Agents represent 9-11-year-old children. In each data zone, agents are created and assigned one of the residential buildings as a home, and to the school for the residential catchment area. Socio-economic position (divided into 4 levels representing a gradient of household income: AB-high, C1, C2, DE-low) and number of cars in the household are assigned to agents based on the probability distributions specified by census data for the data zone of residence. Gender is assigned to agents with a probability of 50%, female or male.

A social network of friends is created where each agent is assigned with 3–8 best friends among the agents in their school.

Some agents are assigned with formal sport activities (FSA). FSA are organised sport sessions that are led by a sport coach, such as: football, swimming and gymnastics clubs, which take place after school in designated venues. The distribution for the number of weekly FSA sessions per agent were drawn from secondary survey data (Scholes and Mindell, 2012) and participation in FSA varies among agents; those from a higher SEP participate on average more times in FSA compared to agents from a lower SEP.

Agents are also assigned with attributes reflecting their *tendency to be active* (*A*) and *preference to play outdoors* (*O*). These attributes reflect heterogeneity within the population and since the exact distribution is unknown to us, we simply assume a normal distribution with mean 1 and variance 0.3^2^ (see supplementary material.2 for sensitivity analysis of these parameters). *A* and *O* are randomly assigned to agents and determine how the agent “behave” compared to others in the population. For example, given the same activity and the same duration, agents with *A* < 1 will accumulate less MVPA compared to agents with *A* > 1. In the same way, given an opportunity to engage in outdoor play and the same environment conditions, agents with *O* < 1 are less likely to participate compared to agents with *O* > 1.

#### 2.4.1 Daily activities

In what follows we present the equations and the parameters that drive agents’ actions and behaviours. The values assigned for these parameters in the simulation and reference to their source are presented in Table 1.

**Table 1:** Parameters’ values of the baseline scenario.

**Parameter**	**Description**	**Value**	**Source**
*F*_*as*_	Frequency of outdoor play on the route home from school, times/weekdays	2/5, average duration 70^&^ min	Frequencies were calibrated to fit aggregated data from UK time-use survey (Office for National Statistics, 2018)
*F*_*friend*_	Frequency of friends meeting, times/weekdays	2/5, average duration 70^&^ min, 50% of meetings outdoors (*γ*)	
*F*_*neigh*_	Frequency of outdoor play in neighbourhood, times/weekdays	1/5, average duration 70^&^ min	
*F*_*shop*_	Frequency of shopping, times/weekdays	1/5, average duration 30^&^ min	UK time-use survey (Gershuny and Sullivan, 2017)
School schedule	Daily 09:00–15:00	lessons - 320 min, recess - 40 min, PE - 60 min, 1/week	Based on a school day in Glasgow
*δ*	Impact of friends - proportion of attribute *A*_*i*_ modified by friends	0.3, Sensitivity analysis explores the range {0–1}	Assumed
*λ*	Influence of presence of others on probability to play outdoors	0.1, Sensitivity analysis explores the range {0–0.6}	Assumed
*s*	Impact of deprivation level on probability to engage in outdoor play	*s* value corresponds to 5 deprivation levels: (least deprived-1, 0.9, 0.8, 0.7, 0.6-most deprived)	Assumed
& Duration is sampled from an exponential distribution with the specified average			

**Fig. 2:**
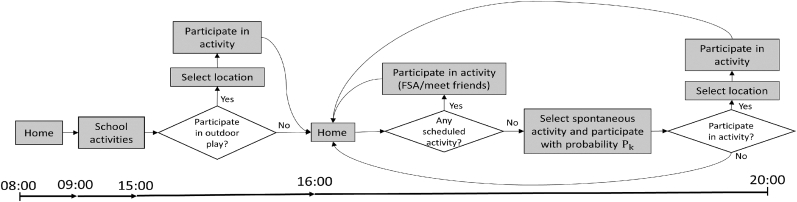
Agent's daily activity procedure.

The agents follow a daily schedule of activities, progressing in a time step representing 1 min (Fig. 2). Each day starts as the agents travel to school. The school day takes place between 09:00–15:00 and includes school lessons, recess and physical education lessons. After school ends, the activities of agents diverge based on pre-scheduled activities such as FSA or meeting friends, and also according to activities that are spontaneously selected by the agent.

**Fig. 3:**
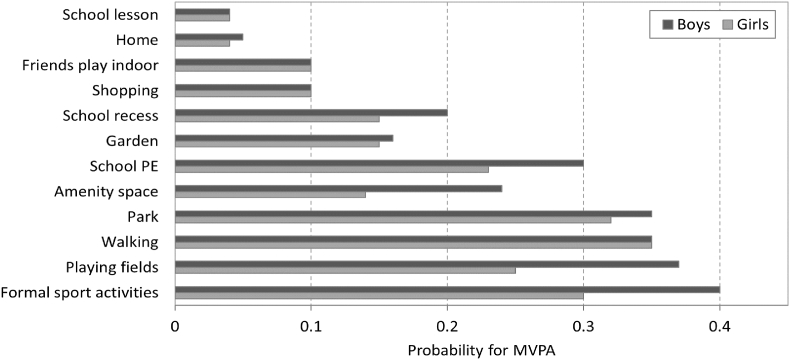
Probability to perform MVPA by land use, for boys and girls. Probability estimates are based on analysis of 109 children residing in urban environments in Scotland: data from SPACES research project (McCrorie et al., 2018).

The first activity takes place directly after school ends, where agents may select to participate in outdoor play on the way home. We assume that agents who are driven home or have a scheduled FSA are significantly less likely to stop on their way home and participate.

Let *P*_*as*,*i*_(*t*) denote the probability of agent i to play outdoors after school at time t:

(1) $$P_{as,i}\left(t\right)=F_{as}\times O_{i}\times s_{i}\times m_{i}\left(t\right)$$

Where *F*_*as*_ is average frequency of playing after school; *O*_*i*_-the preference of agent i to play outdoors,*s*_*i*_ - the impact of the neighbourhood deprivation. $m\left(t\right)=\frac{1}{3}$ if the travel mode is a car or if FSA is scheduled for that day and *m*(*t*) = 1 otherwise. For a sensitivity analysis for the impact of m(t) see supplementary material.2.

After arriving home, agents who have a scheduled FSA or are meeting a friend, travel to the activity. An agent who has no scheduled activity or has completed a scheduled activity, may then select to participate in: outdoor play or shopping. These activities are triggered by the spontaneous activity selection procedure (Fig. 2). This procedure is implemented 4 times per hour (every 15 min on average) after the return from school and until the day ends. The agent randomly selects either outdoor play or shopping and decides whether to participate with probability based on the activity's weekly frequency and modified by conditions in the environment and the preference of the agent. If the activity is not triggered the agent will stay at home. Once an activity is selected by an agent, the duration of the activity is determined by sampling from a time distribution specified for the activity (Table 1). At 20:00, the day ends and the next day begins.

The probabilities for selecting activities after arriving home are calculated as follows:

**Playing outdoors** - To reflect the impact of the close surroundings around the agent's home on the decision to play outdoors, two contributing factors are included: neighbourhood deprivation level and the number of other agents playing outdoors in the vicinity of the agent's home. The presence of other agents playing in the vicinity (300-m radius) is assumed to increase the willingness to engage in the activity, while a higher level of deprivation in the neighbourhood reduces it. The probability *P*_*neigh*,*i*_(*t*) of agent i to participate in an outdoor play at time t is:

(2) $$P_{neigh,i}=F_{neigh}\times O_{i}\times s_{i}\times \left(1+N_{a}\left(t\right)\times \lambda \right)$$

Where *F*_*neigh*_ is a frequency (times per week), *O*_*i*_ - preference of agent i to play outdoors, *s*_*i*_ - impact of the neighbourhood deprivation level; *N*_*a*_(*t*) - the number of agents playing in vicinity of agent i's home at time t and *λ* is their influence. The influence of other agents takes place only when at least 3 agents are present, otherwise *λ* = 0.

**Meeting a friend**: After school, agents coordinate a time for meeting with a friend with weekly frequency *F*_*friend*_. Agents can meet with friends who have no scheduled FSA and are not invited to another friend on the day. It is possible that one agent will host several friends. Before meeting, the agents decide whether to meet at the home of the host or play outdoors in the neighbourhood. Play outdoors takes place with probability *P*_*od*_:

(3) $$P_{od}=\gamma \times \bar{O}\times s_{h}$$

Where *γ* is the proportion of meetings that takes place outdoors, $\bar{O}$ is the average preference of the agents who meet to play outdoors, *s*_*h*_ is the impact of deprivation in the neighbourhood of the hosting agent h.

**Shopping**: One of the activities reported in the UK time use survey by 10% of the children (9–11 years old) was shopping and visiting restaurant and cafes. This frequency was similar to visiting a sport facility (9%) and parks (11%) (Gershuny and Sullivan, 2017). Therefore, we included this activity in the model. Shopping activity is selected with frequency *F*_*shop*_ and is associated with MVPA probability as specified in Fig. 3. The agent selects one of the shops in a range of 1000 m from home, where shops within denser shopping areas are more likely to be selected. Then, the agent selects the travel mode and travel to the selected shop. The duration of time spent shopping is sampled from the time distribution of the activity (Table 1).

#### 2.4.2 Selecting a site for an activity

The sites of FSA are assigned to the participating agents at the initialisation of the simulation. These activities take place in leisure centres, sport fields and schools, located within 1.5 km of the agent's home. Agents with multiple FSA may attend multiple venues during the week.

When participating in other outdoor activities, agents select a site for them. They consider the following types of sites: gardens, sports fields, public parks and spaces designated as residential amenities. When selecting a site for outdoor play after school, agents only include sites that do not extend the route home by more than 500 m (≈15 min walking).

To select, agents assign a rank to each site based on three components and the following weights: distance (60%), area of the site (20%) and the number of other agents visiting the location (20%). As suggested by the literature, we assume that distance is a salient component (Koppen et al., 2014; Dunton et al., 2014; Van Hecke et al., 2016); an increase in distance reduces the rank of a site, while increased presence of other agents increases the rank. Sites with a large area receive a higher rank. The rank R of site j is calculated as follows:

(4) $$R_{j}=0.6\times e^{-d_{j}\times \alpha }+0.2\times e^{-v_{j}^{-1}\times \beta }+0.2\times a_{j}$$

Where *d*_*j*_ is the distance to site j, *v*_*j*_ - number of other agents at site j (*α* = 700^−1^ and *β* = 0.7 are coefficient controlling the impact). *a*_*j*_ = 1 if area >10,000 *m*^2^ and *a*_*j*_ = 0 otherwise.

After establishing the ranking, the agent first considers the site with highest rank. The probability of selecting site j equals the rank of j relative to the rank sum of all other considered sites. If the site is not selected the next highest rank is considered and so on, until a site is selected.

#### 2.4.3 Physical activity

Since this is a simulation, PA levels of each agent can be monitored at each moment and totalling the PA the agents accrue, both as individuals and as a population is straightforward. In the model, the intensity of PA at any one time is associated with the type of activity and its site. Sites are assigned with a parameter that determines the average fraction of time agents perform MVPA while engaging in the activity at the site (Fig. 3). The MVPA fraction for different land use and facilities was estimated based on an analysis of MVPA data collected from 109 children (aged 10–11 years old) living in Edinburgh (50%), Glasgow (30%), Dundee (8%), Aberdeen (12%), as part of the SPACES research project (McCrorie et al., 2018) (socio-demographic characteristic of the sample are presented in supplementary materials .2). These values were used as the probabilities of the agents to perform MVPA when engaging in activities at these sites. The MVPA probability is further modified by the agent's tendency to be active (*A*). At each moment t, agent i may perform MVPA with probability *P*_*MVPA*,*i*_(*t*):

(5) $$P_{MVPA,i}\left(t\right)=A_{i}\times P_{MVPA,k}$$

Where *A*_*i*_ is the tendency of agent i to be active, and *P*_*MVPA*,*k*_ is the MVPA probability associated with land use type k (where agent i is located), as specified in Fig. 3. The accumulation of minutes of MVPA during the day is constantly updated for each agent. When meeting with friends and during the school day we assume that engaging in PA is influenced by friends. Therefore, the tendency of the agent to be active (*A*_*i*_) is partially adjusted to the average tendencies ($\bar{A_{f}}$) of friends: $A_{i}=\left(1-\delta \right)\times A_{i}+\delta \times \bar{A_{f}}$

Where *δ* is a proportion of the agent's tendency that is adjusted.

#### 2.4.4 Travel

When travelling to school, agents either walk or travel by car. The frequency of walking to school is a function of distance to school and street walkability score. The travel mode frequency function is based on an ordinal logistic regression (see regression results in (Almagor, 2021)) estimated from empirical data from 713 children living in Scotland who reported their mode of travel to school for a period of a week (Macdonald et al., 2019). When travelling to destinations other than school, agents calculate a probability to walk *P*_*walk*_ based on distance to destination, walkability score and number of cars in the household. Walking time is explicitly modelled as agents move on the links of the road network, and MVPA minutes are accumulated given a probability specified for walking (Fig. 3).

### 2.5 Model implementation

#### 2.5.1 The baseline scenario

**Fig. 4:**
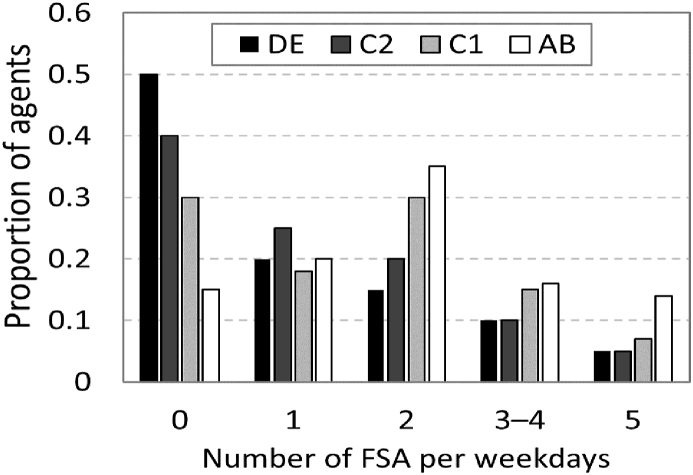
Participation in formal sport activities (FSA) by SEP. On average agents from the higher SEP (AB) participate twice as much in FSA compared to those in lower SEP (DE).

We simulate a baseline scenario using the parameters given in Table 1. In this scenario, school activities correspond to a typical school day schedule in Glasgow. The distribution of FSA in the agent population, as presented in Fig. 4, reflects survey findings regarding the association between SEP and participation in FSA (Scholes and Mindell, 2012). The frequency of after-school activities was calibrated to match population statistics reported in the UK time-use survey (Office for National Statistics, 2018a). The duration of activities is based on average duration reported by individuals in the survey. Since the UK time-use survey reports time use based on one day, we had to determine the weekly frequencies of the activities. To establish the activities weekly frequencies, we simulated multiple combinations of *F*_*as*_, *F*_*friend*_ and *F*_*neigh*_ and selected the combination that generated the best fit of average daily time-use as reported in the data published in the UK time-use survey (Office for National Statistics, 2018). In the baseline scenario, agents spend on average 46 min/day in activities that include outdoor play and FSA, which is in line with the UK time use survey where children spent 50 min/day on average engaging in these activities (Office for National Statistics, 2018).

#### 2.5.2 Experimental design: scenarios of interventions

The ABM provides a ‘virtual lab’ where we can conduct experiments on the agent population. Our experiments are represented by scenarios that explore the potential outcomes of interventions that lead to a change in the PA behaviour of agents. The model does not suggest how such an intervention can achieve the behavioural change in the population, rather it demonstrates the potential impact on MVPA levels when the intervention does work. We focus on three possible domains for interventions: *outdoor play* in the neighbourhood, *school PE* and *active travel*. We created 7 intervention scenarios (S2–S8) by modifying the global parameters that control the frequency of engagement in these activities, and we examined the impact on PA of the population (Table 2). The rest of the parameters remain fixed as described in the baseline scenario (Table 1). To compare between the scenarios, we measure both the mean daily MVPA minutes in the population and the percentage of agents that did not exceed an average of 60 min/day of MVPA, as recommended by PA guidelines (Foster et al., 2019). We also present the impact of the interventions by SEP. Since the simulation includes stochastic elements, each scenario ran for a period equivalent of 30 days and repeated 10 times; results are based on the average of these runs.

**Table 2:** The simulated intervention scenarios.

**Scenario**	**Intervention description**	**Modified parameters**
S2	Increase in the frequency of engagement in **outdoor play** across the neighbourhoods	*F*_*neigh*_ = 2/5
S3		*F*_*neigh*_ = 3/5
S4	Additional daily **PE in all schools**	+30 min of daily PE
S5		+60 min of daily PE
S6	Promotion of **active travel**	All agents walk to school
S7		All agents walk to all activities
S8	**Combination of domains**: - increase in outdoor play - additional PE in schools - active travel to school	*F*_*neigh*_ = 2/5 + 30 min of daily PE All agents walk to school

## 3 Results

### 3.1 Validation of the baseline scenario

**Fig. 5:**
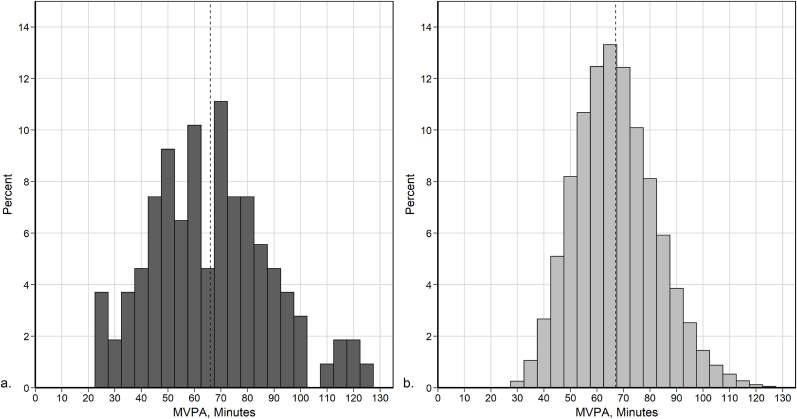
Comparison of ABM outputs with empirical data. Distribution of average MVPA minutes per weekday: (a) data collected from 109 children residing in 4 cities in Scotland. (b) ABM output for 20,770 agents. Dashed line marks the average.

The distribution of daily average MVPA minutes of 20,770 agents, generated by 10 simulations of the baseline scenario is presented in Fig. 5b. To validate the output of the baseline scenario, we compared it to empirical MVPA data collected in the SPACES study (McCrorie et al., 2018) (Fig. 5a).

Agent's MVPA levels over the week are not stable and tend to fluctuate from day to day given the activities and daily schedule of the agent. For most agents, daily MVPA vary by 15%–30% from the average (for further details see: supplementary material.2).

The average daily MVPA from the SPACES data was 66 min (SD 22), compared to 67 min (SD 15) for the ABM (Fig. 5). Based on a two-sample K–S test the modelled MVPA distribution is not significantly different from the distribution of the empirical data (D = 0.12, p = 0.14). In addition, only 14% of the agents accumulated at least 60 MVPA minutes in every weekday, similar to 11.% of the children as reported in the SPACES study (McCrorie et al., 2018). Given that the baseline scenario generates plausible PA outputs, we next explored the impact of intervention scenarios.

### 3.2 Scenarios of intervention

**Fig. 6:**
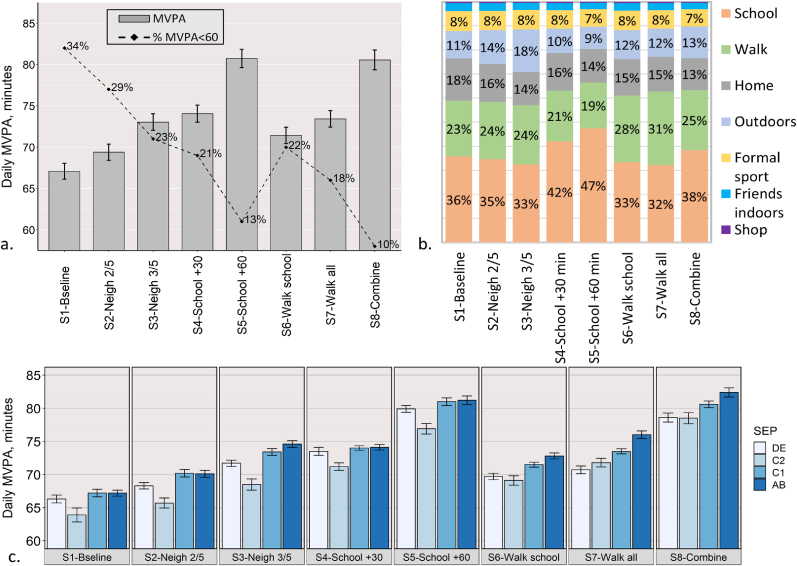
Daily average of MVPA by scenario. (a) Daily average of MVPA minutes in the population (bars and 95% CI) and percent of agents with average MVPA <60 min (dashed line). (b) Percentage of total population MVPA by type of activity. (c) Daily average of MVPA minutes and 95% CI by SEP (AB – high, C1, C2, DE – low).

Fig. 6a presents the average MVPA min/day for each of the scenarios and the percentage of agents accumulating less than an average of 60 min/day of MVPA and, therefore, not meeting the PA recommended guidelines. 95% confidence intervals based on 10 simulations per scenario are also presented.

In the baseline scenario, the average daily MVPA was 67 min and 34% of the agents did not exceed an average of 60 min/day. The largest increase in PA relative to the baseline was achieved for scenario S5 in which an hour of daily PE was added during the school day. In this scenario the percentage of agents that did not accumulate an average of 60 min/day of MVPA was reduced to 13%. Increased frequency of outdoor play in the neighbourhood (S2, S3) increased the average daily MVPA to 69 and 73 min, respectively, and reduced the percentage of agents not exceeding 60 MVPA min/day to 29% and 23%, respectively.

It is worth noting that although the frequency of outdoor play increased linearly by 1 day (S2) and 2 days (S3) in comparison to the baseline, average MVPA levels increased non-linearly by 2 and 6 min. This can be explained by the increased presence of agents playing outdoors that triggered a positive feedback, further encouraging other agents residing in the neighbourhood to engage in outdoor play, which in turn contributed to additional accumulation of PA each day (see: Sensitivity analysis of agent's interactions). In these scenarios, we also measured an increase in the average walking time of agents compared to the baseline. This demonstrates that increased engagement with outdoor play also increased active travel, which, in turn, further enhanced MVPA levels.

In the scenarios where all agents were walking to school (S6) and walking to all activities (S7), the average MVPA increased to 71 and 73 min/day, respectively. The combined scenario (S8), where several activity domains were modified in conjunction, generated an average of 80 min/day of MVPA, with the highest reduction in agents not accumulating an average of at least 60 min/day of MVPA.

As can be seen in Fig. 6c, in the baseline scenario PA varied between SEP groups, where agents from the C2 group accumulated on average the least MVPA/day and agents from the higher SEP groups (C1 and AB) the most. Interestingly, the intervention scenarios impacted the SEP groups differently, where some groups benefited more than others in terms of MVPA levels. For example, in the intervention that increased outdoor play (S3), the highest SEP group (AB) increased MVPA levels more than any other group. In the school interventions (S4–S5) the increase in MVPA levels was similar in all groups and the balance between the groups remained the same as in the baseline scenario.

The scenarios that were focused on active travel (S6–S7) resulted in MVPA outcomes that followed SEP gradient, where the highest SEP group (AB) accumulated the highest MVPA min and the lowest SEP group the lowest (DE).

In all scenarios, most of the daily MVPA of the population was accumulated during school (32%–47%), followed by walking (19%–28%). Accumulation of MVPA in FSA and outdoor play comprised 16%–26% of total MVPA and 13%–18% were accumulated at home (Fig. 6b).

In the ABM we defined behaviours for agents at the individual level, as well as the values of multiple parameters that control their decisions and actions in response to different social and environmental conditions. Only when the simulation is executed, the complex interplay between multiple parameters, interactions of agents and interactions with the environment, are at play and give rise to the effects of the parameters within the PA system. These effects cannot be deduced from the individual level behaviour assigned to individual agents; therefore, in that sense, they emerge from the simulation. The correlations between agents’ characteristics and their MVPA outcomes in the different simulation scenarios provides an indication to the extent of influence that variables have on MVPA accumulation. This is akin to understanding the relative contributions of different components of the PA system and how they change and interact in the different scenarios.

Table 3 presents Pearson r correlation coefficient between the average MVPA min/day accumulated by the agent and several of the agent's characteristics for the simulated scenarios (S1–S8). These correlations provide an indication of the extent of influence of these factors on agents' PA in the different scenarios. This is akin to understanding the relative contributions of different components of the PA system and how they change and interact in the different scenarios. The tendency of an agent to be active (*A*) had the strongest correlation (r = 0.68–0.75) with MVPA in all scenarios, followed by walking time (r = 0.46–0.56). Number of FSA showed a weaker correlation in most scenarios (r = 0.24–0.29), but significantly increased (r = 0.38) in scenario S7 when walking to all activities was the main intervention. This highlights an additional benefit to agents who are participating in FSA that is gained when they actively travel to the activity; that further contributes to increased PA. Number of cars in the agent's household is negatively correlated with MVPA for scenarios S1–S5. This correlation significantly weakened for the scenarios S6–S8; this is expected, since all the agents walk to school in these scenarios and the car is not considered as a transport mode for the school route. The correlation of outdoor play time with MVPA is low (r = 0.12) for the baseline scenario, though it increased to 0.27 in S3 when the frequency of outdoor play in the neighbourhood was increased.

**Table 3:** Pearson correlation coefficient for daily MVPA minutes and agent's attributes.

***♯***	**Scenario**	**Tendency to be active (*A*)**	**Preference to play outdoor (*O*)**	**Walking time**	**Number of FSA**	**Outdoor time**	**SIMD**	**Number of cars**
S1	Baseline	0.68	0.09	0.56	0.29	0.12	0.07	−0.24
S2	Neigh 2/5	0.69	0.12	0.53	0.27	0.16	0.10	−0.27
S3	Neigh 3/5	0.68	0.17	0.51	0.24	0.27	0.20	−0.21
S4	School +30 min	0.71	0.08	0.51	0.26	0.11	0.05	−0.28
S5	School +60 min	0.74	0.07	0.46	0.26	0.09	0.05	−0.24
S6	Walk school	0.72	0.09	0.46	0.32	0.05	0.11	−0.02
S7	Walk all	0.70	0.10	0.51	0.38	0.02	0.12	0.02
S8	Combine	0.75	0.12	0.40	0.26	0.09	0.14	−0.04

The deprivation level in the agent's neighbourhood (SIMD) and the preference of the agent to play outdoors (*O*) are attributes that impact agents' decision to engage in outdoor play. Both these attributes show an increased correlation with MVPA as the frequency of outdoor play is increased (S2–S3). This suggest that in the simulated interventions where outdoor play is promoted, the deprivation level in the neighbourhood and the preference of the agent become more prominent factors that contribute to the variation of MVPA levels between agents. For example, in S3 deprivation level in the neighbourhood contributes more (compared to other scenarios) to the variance in MVPA between residence of high and low deprivation areas.

### 3.3 Sensitivity analysis of agent's interactions

**Fig. 7:**
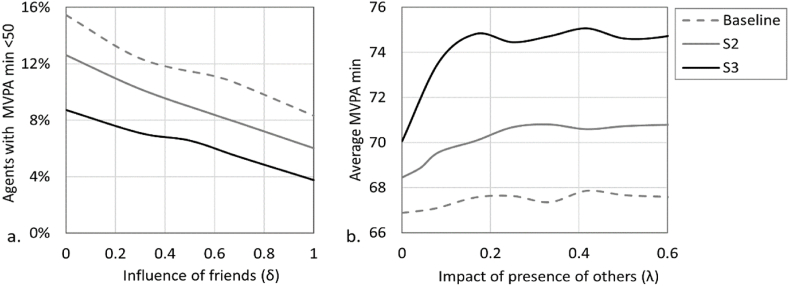
Sensitivity analysis for agents' interactions. a) Proportion of agents with MVPA min <50 (y-axis) for varying values of influence of friends *δ* (x-axis). b) Average MVPA min (y-axis) for varying values of the impact of presence of others *λ* (x-axis) on agents' probability to engage in outdoor play.

In the model, agents interact in two main ways: a) adapting a portion (*δ*) of their tendency to be active (*A*) to the tendency of their friends when engaging in activity together; and b) agents are more likely to engage in outdoor play when other agents are present in the vicinity of their home. Both interactions are supported by research studies as described in the methods section (Macdonald-Wallis et al., 2012; Maturo and Cunningham, 2013; Stearns et al., 2019; Floyd et al., 2011; Pedroni et al., 2019). With that, it is hard to quantify these influences. Therefore, in addition to the values we used in the simulations for these parameters, we explore how changing their values affect the outcomes of the model. For (*δ*) we explore the range 0–1, from no adaptation to full adaptation; for the impact (*λ*) of the number of other agents on probability to engage in outdoor play we explore the range 0–0.6, from no impact and up to 60% impact for each agent that is present outdoors. We test these parameters for three scenarios: baseline, S2 and S3 (Fig. 7).

As can be seen in Fig. 7a, as the influence of friends increases (*δ*), we observe a reduction in the percentage of the less active agents (MVPA <50 min). This means that this type of interaction between agents contributes to reducing the levels of the least active in a population, and as this influence is further strengthened, these levels are further reduced.

As for the impact of others on agent's engagement in outdoor play (*λ*), in all three scenarios, as the impact increases, the average MVPA in the population increases too, until it flattens around the values of 0.3–0.4. It is also apparent that in scenarios S2 and S3 where the frequency of outdoor play in the neighbourhood is higher, the impact of others leads to a sharper increase in MVPA. The intervention scenarios S2 and S3 increased the engagement of agents in outdoor play; the increased presence of agents outdoors following the intervention further attracted more agents living in the vicinity to join, triggering a positive feedback effect. As can be seen in Fig. 7b, the positive feedback is the strongest in S3. This suggests that the intervention helped to trigger the feedback that was much weaker in the baseline scenario, and MVPA levels were further enhanced by this process.

## 4 Discussion

In this study we presented an ABM that explored children's PA in an urban setting by implementing social-ecological theory principles within a complex systems method. PA levels were generated by explicitly simulating agents' activities and interactions with each other and with the physical environment. The ABM was validated with empirical data and generated a realistic distribution of daily MVPA minutes.

The most important characteristic in influencing PA levels was found to be the agent's tendency to be active (*A*). This characteristic defined how physically active the agent tends to be relative to others in the population when engaging in a given activity. Therefore, it is expected that this characteristic will correlate with MVPA levels. With that, what did emerge in the simulation was the *extent* to which this characteristic influenced the accumulation of MVPA in comparison to other characteristics that vary among agents (such as: number of FSA, outdoor play time, active travel, home location and preferences for outdoor play). It shows that the individual level is the most influential level in the modelled PA system. Further analysis of the impact of parameters (*A*) and (*O*) on the distribution of MVPA in the population is provided in a sensitivity analysis (see: supplementary material .2). This characteristic can be interpreted as an abstract psychological characteristic, combining multiple psychosocial constructs such as self-efficacy, motivation, and personality, that affects how active a child is in relation to others. The importance of self-efficacy in determining PA levels of individuals was demonstrated in multiple studies and is found to be a strong predictor for PA (Manley et al., 2014; Hamilton et al., 2017; Banks et al., 2017). While our model was not intended to unpick the salient latent factors underlying our ‘tendency’ construct, it corroborated the centrality of individual level physiological characteristics in influencing PA levels. Interventions that are focused only on increasing the exposure to an activity, with no support for individuals who have internal barriers, will be less effective in increasing PA.

While we assumed that agents’ tendency to be active (*A*) is independent of preference to be outdoors (*O*), it is possible that these behavioural parameters reinforce each other, and, therefore, may further increase or decrease PA. The existence and implication of correlation between these parameters requires further exploration by the PA research and are beyond the current scope of our research. In this case, the model building process raises issues that are still unexplored, and, this in itself, is an added value of ABM.

The second most important factor for PA was the walking time of the agent. In all scenarios, MVPA accumulated by walking was between 19% and 31% of the total MVPA accumulated by the agent population. This highlights the importance of creating an infrastructure that supports active travel along routes frequently used by children, such as: wide sidewalks, controlled road crossings, zones of reduced traffic and controlled speed and streets closed for vehicle traffic (Nevelsteen et al., 2012; Ikeda et al., 2020; Bosch et al., 2020).

On the contrary, engagement in shopping provided very low contribution to overall MVPA. This suggests that encouraging children to substitute time spent in shopping with other activities that contribute more to MVPA is a possible route for an intervention.

Multiple studies have found an association between neighbourhoods' characteristics and PA (Davison and Lawson, 2006; Ding et al., 2011; Villanueva et al., 2016). Understanding how PA varies across the urban environment is beneficial for policy makers to design and deliver PA interventions for the most needed areas, especially when resources are scarce. Unfortunately, collecting PA data on a fine geographical scale covering an entire city is costly, hard to implement and is yet to be achieved to the best of our knowledge. ABMs can be used as an alternative pathway for generating synthetic data on PA for policy evaluation. Some studies suggest methodologies to support the design of a conceptual framework of ABMs for PA. These include involving a diverse expert-based panel for a model's concept assessment (Garcia et al., 2017); a participatory method for engagement with youth to establish the agents' decision rules (Frerichs et al., 2020); and a general ABM framework for simulating non-communicable disease (Aziza et al., 2016). Our study adds to this work and demonstrates how theoretical concepts can be integrated with empirical data and findings from the broader literature to simulate children's PA by means of an ABM with explicit representations of the urban settings.

Using the model, we explored the potential effect of several interventions on children's daily MVPA. We found that all simulated interventions could potentially be useful in increasing PA and the percentage of children meeting the recommended PA guidelines, but with varying effectiveness and with differing impacts on SEP groups.

The ‘outdoor play in the neighbourhood’ scenario demonstrated that increasing the frequency of outdoor play contributed to population PA beyond the direct engagement in the activity itself. The findings demonstrated that outdoor play may enhance PA by increasing walking and by creating a positive feedback where the presence of children playing outdoors further attracts others residing in the vicinity to engage in the same activity. This is in line with previous research which has shown that promotion of active travel, neighbourhood relationships and child friendliness were positively associated with outdoor play (Lee et al., 2021). This suggests that a possible strategy for increasing PA is to create ‘catalyst’ events (such as: community get-togethers, street closure events) at focal points in the neighbourhood that could attract an initial crowd of children and potentially trigger a positive feedback that enhance engagement with outdoor play.

Findings from the simulations that promoted active travel indicated that these would benefit more children from a higher SEP, as they are more likely to have a car in their household and more likely travel to additional after school activities, such as sport clubs and leisure centres. While walking is a central component for children's daily MVPA, a policy intervention that focuses solely on active travel may lead to increasing inequality in PA among SEP groups. Active travel interventions in primary school age groups have been shown to be effective in increasing active commuting, particularly those involving walking school buses and educational strategies, with effect sizes not appearing to be associated with the complexity of the intervention (Jones et al., 2019). The implication being that even simple active travel interventions may be successful and scalable.

In the school-based intervention scenario, additional PE lessons during school hours, had a substantial impact on the population average MVPA, since all children were equally exposed to the intervention daily. In the model, we assumed an effective delivery of the PE lessons and exposure of all pupils in all schools. However, in a real school setting such an intervention would require schools to allocate time for and assign trained staff members to deliver PE lessons effectively. It is likely that when such an intervention is implemented on a city scale the quality of implementation will vary between schools and may reduce the impact of the intervention when observed at a population level (Buchan et al., 2012; Kriemler et al., 2010; Jaakkola et al., 2017).

It is important to note that any individual approach, such as school-based PA interventions, may not be effective at increasing children's overall daily time spent in MVPA, as children may adjust their behaviour and be less active during other parts of the day (Møller et al., 2014; Love et al., 2019). Therefore, multiple interventions across settings might be required. With that, our ABM does not account for adaptation and compensation of PA behaviour by agents as a response to an increase in PA in a particular domain. Whether such adaptation processes occurs in children is still debatable. Goodman et al. (2011) found no evidence for PA compensation for a range of activities including: active travel, school PE, games and after school structured sports. In addition, no evidence was found for compensatory increases in PA as a response to reduced opportunities of PA in school (Dale et al., 2000; Morgan et al., 2007). Conversely, literature does exist to support the existence of behavioural compensation. Ridgers et al. (2014) for instance, were able to demonstrate that every additional 10 min of MVPA on a given day would result in 5 min less MVPA the following day. Møller et al. (2014) found no differences in overall PA levels between children attending sports schools and normal schools. Sports schools children were more active than normal schools children during school time, but less active during leisure time. Though, the study does not infer on causality of the observed PA compensation; it doesn't conclude whether the compensation is a result of children adapting their behaviour, or, parents restricting their children's after school PA. The potential impact of an intervention in multiple settings is exemplified in the simulation of combined interventions (S8). This simulated intervention resulted in the largest percentage of agents meeting the PA guidelines. It demonstrated that an intervention in a diverse population is more likely to impact the least active when it operates in several domains of the children's day and offers diverse opportunities to engage in PA. In addition, when the intervention is implemented in several domains, the PA “dose” in each domain can be reduced, making the intervention more likely to be achieved and possibly maintained.

We also found that the interaction in which agents adapted their PA behaviour to the behaviour of their friends reduced the percentage of agents who are the least active in the population. Our sensitivity analysis revealed that as the influence of such interaction increased the percentage of the least active reduced. This implies that encouraging children to be active in diverse groups (in terms of PA level), while being supportive of each other, will likely have a positive effect on the least active. This speaks specifically to our theoretical understanding of the role social support and social norms may play when integrated into a wider systemic approach to behaviour change (Draper et al., 2015; Lee et al., 2020).

A key strength of our approach was that the model produced an empirical representation of the complexities of our theories about PA, which cannot be done using reductionist methods. The model can simultaneously speak about agents’ differences, inequalities and overall population outcomes. In a sense, the model allows us to test our understanding of the ways in which children accrue PA in their day and explore the influence of their interactions with each other and their environment. The model generated levels and distribution of PA that were similar to what is observed in reality, and, therefore, provided a plausible description of the PA system. The approach also enabled insights and observations about the possible outcomes of interventions without the need for costly field work and lengthy experimental procedure.

However, our ABM also has several limitations: It is focused on MVPA as a measure for PA intensity and does not include additional PA components such as muscular strength, flexibility and motor skills. While it is likely that children's willingness to engage in PA is affected by the quality and features of sites available to them in their neighbourhood and at school, due to the lack of data, these were not considered in the decisions of the agents. For example, all park sites were perceived as similar by the agents with no effect of features such as amount of greenery, trees, or maintenance conditions; and all school environments were conceptualised as similar. Variation in quality of these features of the built and natural environment will influence the outcomes of interventions, I.e., some will be more feasible to implement than others and could relate to considerations such as physical space and available funding. Where we assumed universal exposure to certain intervention, we also assumed maximal fidelity related to the delivery of certain interventions (i.e., additional school-based PE time or all children engaging in an active commute). In reality, this will not be the case, and as such, the population effect will likely be attenuated. Future work may wish to model variation in the delivery of chosen interventions to explore its impact on PA outcomes.

Beyond modelling variation in the fidelity of any particular intervention, the model could be further adapted to better represent children's behaviours if more research is conducted using methods of stated preferences to reveal how they choose activities and sites and how PA behaviour is affected (Frerichs et al., 2020). Finally, while the model included a social network and peer influence was modelled, further exploration is required to understand how different types of social networks and alternative ways to model the effect of peer influence may impact PA dynamics; this analysis is beyond the scope of the current study.

## 5 Conclusions

The ABM we developed demonstrated how children's PA can be represented and explored as a complex system, where the influences and interactions of multiple levels (individual, social and environment) are represented. Using the model, we explored the potential impact of interventions in several activity domains including outdoor play, school PE, active travel and their combination, on increasing population MVPA levels and the percentage of agents meeting PA guidelines. The model also showed that the interventions had a differing impact on SEP groups. Exploring agents' interactions suggested that outdoor events in neighbourhoods can enhance the engagement of children with PA; and that encouraging children to be active in diverse groups will likely have a positive effect on the least active.

We suggest that ABMs should be used more commonly as tools to develop and test PA theories by researchers, to assist interventionists develop and refine their programmes, as well as to support policy makers in exploring the implication of policies. The model building process itself can assist non-modellers to clearly define and sharpen different aspects of a theory, since the ABM requires detailed definition of processes and parameters. ABMs not only challenge the researcher to think in a systemic way and clearly formalise what is known (or assumed) about the system, but they also reveal parts that are unknown and should be further explored in the field. As our understanding of children's PA behaviour and decision-making grows, better ABMs can be developed to assist in exploring the PA system.

## Declaration of interest statement

The authors declare that there are no conflicts of interest.

## Data statement

The ABM code together with a file containing the simulation results generated during the current study are available in the GitHub repository: https://github.com/Jonatanalma/ABM_Children_activity.For a video demonstration of the ABM see: https://www.youtube.com/watch?v=RRvnboWudFk.

## Funding statement

This work was supported by the UK Medical Research Council {grant numbers: MC_UU_12017/10, MC_UU_00022/4, MC_UU_12017/14, MC_UU_00022/1} and Chief Scientist Office {grant numbers: SPHSU10, SPHSU14, SPHSU16, SPHSU19}. The grants provide 5 years of core research support for JA, AM, PM and RM at the MRC/CSO Social and Public Health Sciences Unit, University of Glasgow.

## Authors’ contributions

JA conceived and programmed the model, conducted the simulations, analysed and interpreted the results, and drafted the manuscript. PM acquired the empirical data used in the model. PM and AM advised on the design of simulation experiments. PM, AM and RM critically revised and contributed to the manuscript.

## Declaration of competing interest

The authors declare that there are no conflicts of interest.

## Appendix A Supplementary data

Multimedia component 1 Description of the agent-based model using the ODD + D protocol: Overview, Design Concepts, Details + Agents’ Decision, PDF file. Multimedia component 1

Multimedia component 2 Sensitivity analysis and additional graphs and tables. Multimedia component 2
