# Supplementary material for: How can an agent-based model explore the impact of interventions on children's physical activity in an urban environment?
Source: Health Place. 2021 Nov;72:102688. doi: 10.1016/j.healthplace.2021.102688 (PMC8633766; doi:10.1016/j.healthplace.2021.102688)
Supplement: Multimedia component 1 — Description of the agent-based model using the ODD + D protocol: Overview, Design Concepts, Details + Agents’ Decision, PDF file. [file mmc1.pdf]

How can an agent-based model explore the impact of interventions on children's physical activity in an urban environment?

Overview, design concept, details and agent's decision making: ODD+D protocol

Jonatan Almagor, Anne Martin, Paul McCrorie, Rich Mitchell

1. Purpose

To explore the impact of interactions between individual characteristics, the social environment and the urban environment on children's daily physical activity (PA); and to evaluate the potential impact of interventions in the following domains: outdoor play, physical education in school, active travel and their combination.

2. Entities, state variables and scales

There are two types of entities in the model: The agents and the environment. The agents represent children and the environment represents the geographical urban landscape where agents preform daily activities.

2.1 The environment

The environment provides the physical space within which agents act. It consists of spatially referenced vector layers of land-use, houses, schools, shops, leisure centres and a road network (Figure 1a). Land-use are characterised by type k (e.g. park, playing field, garden) and an attribute $\mathrm{P_{MVPA}}$ that represents the degree to which agents are physically active when engaged in an activity located on land-use type k. $\mathrm{P_{MVPA,k}}$ denotes the average probability of agents to preform moderate-to-vigorous physical activity (MVPA) per unit of time spent on land-use type k.

Roads are used by agents to travel between activities and the distance between sites is based on the road network distance. The environment is divided into zones; these are small geographical areas used in the collection and reporting of administrative and census data in Scotland. The data zones include demographic statistics; the level of deprivation (SIMD) of the area, divided into 5 levels based on quintileand; a street walkability index divided into 5 levels.

2.2 Agents

Agents represents 9-11-year-old children; they are dynamic entities that are mobile and implment a daily schedule of activities during which they engage in PA. Agents are assigned a home and a school in the city. They are characterised by socio-economic status (SES), gender, number of cars in the household and number of weekly formal sport activities (FSA). Agents are also characterised by attributes reflecting their *tendency to be active (A) and preference to play outdoors (O)*. These attributes reflect heterogeneity within the population and since the exact distribution is unknown to us, we simply assume a normal distribution with mean 1 and a variance of 0.3^2. *A* and *O* are randomly assigned to agents and determine how the agent "behave" compared to others in the population.

While preforming an activity or travelling between locations, the agent may perform MVPA. A state parameter counts minutes of MVPA performed by the agent during the day. Agents sense their environment; they have knowledge about availability of land-use and facilities in the vicinity of their home and school and can estimate distance. They are also socially aware and can sense the presence of other agents in their vicinity. They use this knowledge for selecting a site for their activities.

2.3 Representation of space

Space is explicitly represented using geo-referenced vector layers that form the environments (e.g. land-use, houses and roads) (Figure 1a). Agents location in the environment is updated as they move between activities. To select a site for activities and decide on a travel mode, agents evaluate distance and availability of sites (facilities and land-use) in relation to their location. The road network is used by agents to calculate the travel route between activities. The spatial distribution of land-use and facilities in the vicinity of the agents affects their engagement in particular activities, the distance they travel and the mode of travel they use (car vs. walk), and, in turn, it affects the accumulation of MVPA during the day.

2.4 Temporal and spatial resolution

The model simulates agents' activities during weekdays between the hours: 08:00 -20:00. Model cycle represents one minute (Δt=1 minute), and each simulation runs for 30 days.

The spatial extent of the model covers 34 km^2 of the city of Glasgow, (20% of the city municipal area) with 2070 children agents (Figure 1b).

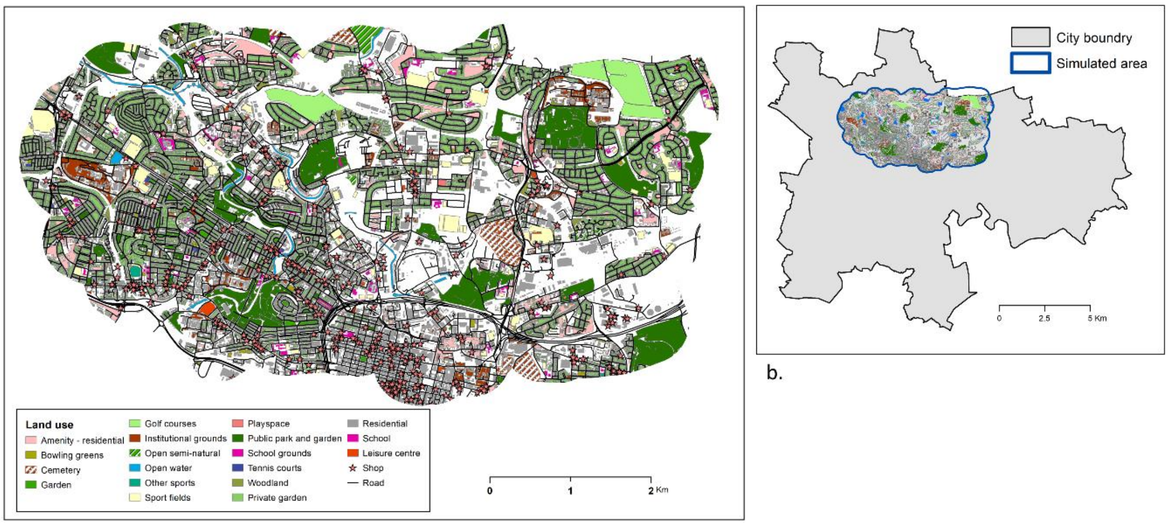

a. b.

Figure 1: The environment. (a) Georefrenced vector layers of land use, buildings and road network form the urban environment. (b) The environment covers 34 km^2 of the city of Glasgow.

2.5 Process overview and scheduling

2.5.1 Daily activities

The agents follow a daily schedule of activities, progressing in a time step representing one minute (Figure 2). Each day starts as the agents travel to school. The school day takes place between 09:00-15:00 and includes school lessons, recess and physical education lessons. After school ends, the activities of agents diverge based on pre-scheduled activities such as FSA or meeting friends, and also according to activities that are spontaneously selected by the agent. The first activity takes place directly after school ends, where agents may select to participate in outdoor play on the way home. We assume that agents who are driven home or have a scheduled FSA are less likely to participate.

After arriving home, agents who have a scheduled FSA or are meeting a friend travel to the activity. An agent who has no scheduled activity or has completed their scheduled activity, may then select

to participate in: outdoor play or shopping. These activities are triggered by the spontaneous activity selection procedure. This procedure is implemented 4 times per hour (every 15 min on average) after the return from school and until the end of the day. The agent randomly selects either outdoor play or shopping and decides whether to participate with probability based on the activity's weekly frequency and modified by conditions in the environment and the preference of the agent. If the activity is not triggered the agent will stay at home. If an activity is selected by an agent, the duration of the activity is determined by sampling from a time distribution specified for the activity. At 20:00, the day ends and the next day begins.

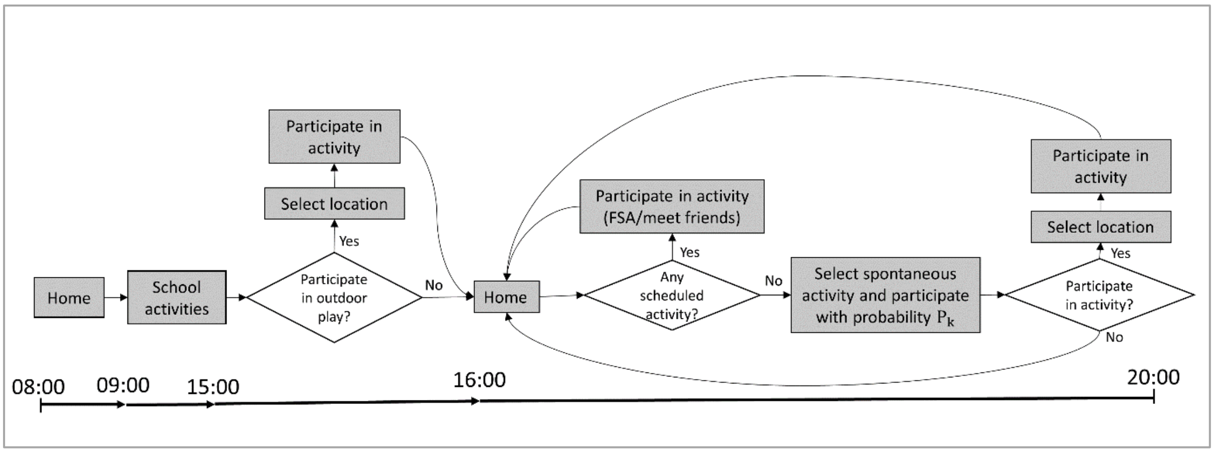

Figure 2: Agent's daily activity procedure.

2.5.2 Travel

When traveling the agent moves along the road-network from her current location to the location of the next activity. Travel is either active (walking) or passive (using a car). Before each travel the mode of travel is selected by the agent. The mode of travel determines the speed of movement (and therefore the duration of travel) and the MVPA that may be accumulated during the travel. During passive travel no MVPA is performed by the agent, while in active travel at each unit of time MVPA may be performed with a probability as specified in Figure 3.

2.5.3 Performance of physical activity

In the model, the intensity of PA at any one time is associated with the type of activity and its site. Sites are assigned with a parameter that determines the average fraction of time agents perform MVPA while engaging in the activity at the site (Figure 3). The MVPA fraction for different land use and facilities was estimated based on an analysis of GPS and accelerometer data collected from 109 children (aged 10-11 years old) living in urban environments in Scotland, as part of the SPACES research project (McCrorie et al., 2018). Accelerometery data from the SPACES study was collected across 7 days using ActiGraph GT3X+ devices, 10 second epoch accumulation, and the Evenson threshold cutpoints to discern time spent in MVPA (Evenson et al., 2008). These values were used as the probabilities of the agents to perform MVPA when engaging with activities at these sites. The MVPA probability is further modified by the agent's tendency to be active (*A*).

3. Design concepts

3.1 Theoretical and Empirical Background

The social-ecological framework provides a theoretical background for the ABM. We assume the PA of an individual is influenced by interrelated factors on multiple levels: **individual, social environment, environment and policy** (Sallis et al., 2006). Factors related to these multiple levels are integrated within the model affecting the agents' decision-making, their activity schedule and behaviour. Collectively, this simulates a complex system from which individual PA and, subsequently, population levels of PA emerge. The influence of each level is theorised and then implemented by drawing on empirical evidence and the literature. Below, we set out the ideas and relationships that are simulated. Then, we show in more detail how these are implemented in the model.

At **the individual level** agents are characterised by socio-economic status (SES) and PA-relevant attributes that reflect economic constrains associated with SES; these include availability of cars in the household and participation in formal sport activities (e.g. sports clubs). Car availability affects the travel mode selection (car vs. walking) and participation in formal sport activities affects the activity schedule of the agent. Both, in turn, affect levels of PA.

As for the intensity level of PA, it differs by agent's gender, as we observed in empirical data (Figure 3). Moreover, psychological theories assert that variation in PA behaviour is the outcome of a complex interplay between psychological variables (Hagger et al., 2001; Rhodes et al., 2019). Although our model does not explicitly represent the levels of psychological variables, we do include an abstract variable representing a 'tendency' to be active that introduces variation between agents: given the same activity, depending on their 'tendency', some agents are more likely to accumulate MVPA while others are less. In addition, we assume that agents differ in their willingness to engage in outdoor activity. This could be because of differing preferences (Cleland et al., 2010) and/or parental influence (Remmers et al., 2014).

The influence of the **social environment** is based on findings suggesting that children adapt PA behaviour in accordance with the behaviour of friends (Macdonald-Wallis et al., 2012; Maturo & Cunningham, 2013; Stearns et al., 2019). Moreover, children may be more attracted to participate in activities when and where other children are present (Floyd et al., 2011; Pedroni et.al., 2019). To implement these influences, social ties are created between agents to represent a friendship network. Agents adapt their tendencies and preferences to those of their friends when performing activities together. In addition, agents sense the presence of other agents in their vicinity and are more likely to participate in outdoor activity when more agents are present.

Evidence suggests that adverse social conditions in the neighbourhood may lead to concerns about safety and discourage activity outdoors (Rees-Punia et al., 2018; Marquet et al., 2019). To reflect this, an agent's likelihood to participate in an outdoor activity in the neighbourhood is affected by deprivation level. Neighbourhoods' deprivation level in the model are based on the Scottish Index of Multiple Deprivation (SIMD) (Scottish Government, 2020). The index is a combined measure of the extent to which an area is deprived across seven domains: income, employment, education, health, access to services, crime and housing.

The urban **environment** provides places to engage in PA behaviour. Land use types differ in the activities they afford and facilitate and are associated with varying intensity levels of PA (Prince et

al., 2019). To represent the impact of land use on intensity level of PA, each type of land use in the model is assigned a parameter that controls the intensity level of PA performed by the agents when they are located there. Land use parameters that control PA were derived from an analysis of empirical data of children's MVPA accumulation at different land use and facilities (Figure 3).

The configuration of the living environment may also affect accessibility to sites for engagement in outdoor activity. Distance is a key consideration for selecting a site for recreational activities (Koppen et al., 2014; Dunton et al., 2014; Van Hecke et al., 2016). In the model, distance to a site as well as the social influence (presence of others at the location) and the size of the site are considered by the agents when they select a location for outdoor activity. Distance and street walkability are central determinants of children's choice for mode of travel (Oliver et al., 2015; Williams et al., 2018; Macdonald et al. 2019), thus affecting PA accumulated by active travel. In the model, roads are explicitly represented allowing agents to evaluate walkability and distance before every journey to decide on the preferred mode of travel. Empirical data from children's travel diaries are used to estimate a probability function used by the agents to decide on the mode of travel (car vs. walk).

In this simulation, **policy** is represented as different intervention scenarios. In the scenarios we manipulate parameters that control the engagement of the agents in specific activities during the week and observe the impact on PA levels in the population. More specifically we test three potential intervention domains in the children's day: school, outdoor play in the neighbourhood and active travel.

3.2 Individual decision making

Before traveling between activities agents decide on a mode of travel (active or passive). Agents first calculate the shortest route to the destination and evaluate the distance and street walkability. Based on these factors a probability for active travel is calculated that is used by the agent for probabilistic choice. As distance increases the probability for active travel decreases, and as walkability increases the probability increases.

Agent's daily schedule includes participation in outdoor play. Agents' decision to participate is derived by an average frequency of the activity (times per week), the presence of other agents outdoors in the vicinity, neighbourhood deprivation level (SIMD index), and the preference of the agent to be outdoors (O).

When agents consider participating in outdoor play directly after school ends, the travel mode and a planned FSA on the day also affect the decision: traveling home by car reduces the likelihood to engage with outdoor play as well as a schhedualled FSA on the day. All the aforementioned factors contribute to the agents' participation probability.

Following a decision to participate in outdoor play, agents select a site for the activity. Sites that are considered by the agent have the following land-use: gardens, spaces designated as residential amenities, sport fields and public parks. To select a site, agents rank available sites based on distance, no. of other agents present at the site, and size of the site. Agents consider optional sites starting from the highest to lowest rank. The probability to select a site reflects the site's rank relative to the other available sites; in this way the "best" site is not always selected by the agents and reflect aspects of bounded rationality.

When friends are meeting, they either play indoors at the home of the host agent or play outdoors. When playing outdoors they select a site as described above.

3.3 Individual sensing

Agents sense their environment; they have knowledge about availability of land-use and facilities in the vicinity (800 m radius) of their home and school and can estimate distance. They are also socially aware and can sense the presence of other agents in their vicinity (300 m radius).

3.4 Heterogeneity

Agents include heterogenous characteristics: gender, SES, no. of cars, no. of FSA, tendency to be active (*T*), preference to be outdoors (*O*). The configuration of the urban environment (land-use, buildings, facilties and roads) surrounding agents' living area differ based on the home and school location.

3.5 Interactions

Agents interact in several ways:

Agents coordinate time to meet with a friend who has no scheduled FSA and is not invited to another friend on the day.

When friends meet, they make a joint decision whether to play outdoors based on the average tendencies of all agents involved.

Agents' tendency to be active is adopted to the tendency of their friends while preforming activity together (during a meeting or at school).

Agents are aware of the presence of others in their vicinity and are more likely to participate in outdoor activity as more agents are present.

3.6 Stochasticity

Agents' decisions for engaging in activities and selecting the site of activity are based on probabilistic choices and therefore include stochasticity. The duration of preforming an activity is stochastic, it is randomly sampled from a distribution before the agent engage in the activity. Performing MVPA per min of engaging in an activity is based on probability.

Initialisation of agents characteristic are based on a sampling procedures (section 4.1).

3.7 Emergence

The emergent property in the model is the daily minutes of MVPA for each agent and the distribution of this property at the population. MVPA distribution can also be compared for different groups of agents (e.g. gender, SES) and at different areas in the city.

4. Details

4.1 Initialisation

Agents are generated for each geographical zone (DataZone) and their attributes are assigned as specified in Table 1.

Table 1: Initalisation process of agetns' attributes.

Attributes	Assignment
Social economic position (SEP)	4 levels: AB-high, C1 ,C2, DE-low<br>Sampled from the SEP distribution in the DataZone (2011 census)
Number of cars	Range (0-2) Sampled from the cars distribution in the DataZone (2011 census)
Gender	Probability of 50% (boy/girl)
Home location	Randomly selected residential buildings within the zone
School	Assigned based on catchment area
Formal sport activities (FSA)	Number of FSA per week range (0-5), sampled from a distribution conditioned on SES based on Scholes&Mindell, 2012.<br>FSA site, day and time:<br>Site- randomly selected from: leisure centre, sport field, school grounds, within 1500 m' from home.<br>Day- one of weekdays; Hour- one of: 16:00, 17:00, 18:00.<br>For more than one FSA, different days are assigned
Preference to be outdoors (O)	Randomly sampled: Normal (mean=1, STD=0.3)
Tendency to be active (A)	Randomly sampled, Normal (mean=1, STD=0.3)
Social ties	Range (3-12) friends.<br>a social network is created: agents iteratively select 3 random agents from school, relations are reciprocal.
Agent's neighbrhood deprivation level	The data zones include the level of deprivation (SIMD) of the area, divided into 5 levels based on quintile (Scottish Government,2020).

4.2 Sub models

4.2.1 Participation in outdoor activity on the route home

At the end of the school day agents may participate in outdoor activity on the way home. We assume that agents who walk home are more likely to participate in outdoor activity compared to those who travel by car and to those who have a planned FSA on the day. Let $P_{as,i}$ denote the probability of agent i to participate in outdoor activity after school:

Let $P_{as,i}(t)$ denote the probability of agent i to play outdoors after school at time t:

$$P_{as,i}(t) = F_{as} \times O_i \times s_i \times m(t)$$

Where F_{as} is the average frequency of playing after school; O_i - the preference of agent i to play outdoors, s_i - the impact of the neighbourhood deprivation. Where $m(t) = \frac{1}{3}$ if the travel mode is a car or if FSA is scheduled for that day, and m(t) = 1 otherwise.

s value corresponds to 5 deprivation levels (SIMD) as specified in Table 2.

Table 2: Impact of deprivation level on probability to play outdoor (s).

Deprivation level	s
1- Most deprived	0.6
2	0.7
3	0.8
4	0.9
5- least deprived	1

4.2.2 Participation in outdoors activity in the neighbourhood

The probability $P_{neigh,i}(t)$ of agent i to participate in an outdoor play at time t is:

$$P_{neigh,i}(t) = F_{neigh} \times O_i \times s_i \times (1 + N_a(t) * \lambda)$$

Where F_{neigh} is a frequency (times per week), O_i - preference of agent i to play outdoors, s_i - impact of the neighbourhood deprivation level; $N_a(t)$ - the number of agents playing in vicinity of agent i's home at time t and λ is their influence (a fraction). The influence of other agents takes place only when at least 3 agents are present, otherwise $\lambda = 0$.

The number of other children agents playing outdoors at time t is measured in a radius of 300 meters around the agent's home.

4.2.3 Meeting a friend

Agents coordinate a meeting with a friend with frequency F_{friend}. The agent selects a friend who has no scheduled FSA and is not invited to another friend on the day; a time is assigned for the meeting. If no friend is available, the meeting will not take place. One agent might be hosting several friends. Before the meeting agents decide whether to stay at the home of the host or play outdoors in the neighbourhood. Agents select to play outdoors with probability P_{od}:

$$P_{od} = \gamma \times \overline{T} \times s_h$$

Where γ is the proportion of meetings that takes place outdoor, $\overline{T}$ is the average tendency of the agents to play outdoors, s_h is the impact of crime levels in the neighbourhood of the hosting agent.

If the meeting takes place outdoor, the host agent selects the site of the activity as described in section 4.3.4 . The duration of the meeting is randomly drawn from a time distribution that characterises the activity.

4.2.4 Shopping activity

Shopping activity is selected with frequency F_{shop}. The agent selects one of the shops in a range of 1000 meters from home. Shops located within denser shopping areas are more likely to be selected. The agent rank shops based on the density (radius of 100 meters); probabilistic choice is implemented, where the probability to select a shop is proportional to the density relative to the sum of densities of the other shops. A duration for the activity is assigned and the agent travel to the shop location.

4.2.5 Formal sport activity

Agents are assigned with FSA they participate during the week. On average, agents from higher SES participate in more FSA per week compared to the ones in lower SES (Scholes&Mindell, 2012). FSA takes place in: leisure centre, playing fields and school grounds. Each FSA is assigned to one of the facilities that are within 1500 meters of the agent's home together with the day and time of the activity. The duration of FSA is one hour as specified in Table 1.

4.2.6 Selection of a site for outdoor activity

When participating in other outdoor activities, agents select a site for them. They consider the following types of sites: gardens, sports fields, public parks and spaces designated as residential amenities. When selecting a site for outdoor play after school, agents only include sites that do not extend the route home by more than 500 meters (~15 minutes walking). To select, agents assign a rank to each site based on three components and the following weights: distance (60%), the area of the site (20%) and the number of other agents visiting the location (20%). As suggested by the literature we assume that distance is a salient component (Koppen et al., 2014; Dunton et al., 2014; Van Hecke et al., 2016); an increase in distance reduces the rank of a site, while increased presence of other agents increases the rank. Sites with a large area receive a higher rank.

The rank R of site j is calculated as follows:

$$\mathrm{R_j} = 0.6\ \times e^{-d_\mathrm{j}\times\alpha} + 0.2 \times e^{-\mathrm{v_j}^{-1}\times\beta} + 0.2 \times a_\mathrm{j}$$

Where, d_j *Is the* distance to site j; v_j - number of other agents at site j ($\alpha\ and\ \beta$ are coefficients controlling the impact); a_j - area dependent parameter: $a_j = 1\ if area > 10{,}000$; $a_j = 0\ if area \leq 10{,}000$.

After establishing the ranking, the agent first considers the site with highest rank. The probability of selecting site j, equals the rank of j relative to the rank sum of all other considered sites. If the site is not selected the next highest rank is considered and so on, until a site is selected.

The agent's selection procedure is as follows:

-calculate the rank for the available sites

-order the sites in a list by descending order of rank

-select the first site s in the list:

$if\ \frac{R_s}{\sum_{k=1}^{n} R_k} > random$, then the current site is selected

if not, repeat the same procedure on the next site in the list

Continue the procedure until a site is selected

The duration of the activity is determined by a random draw from a time distribution that characterise the activity.

4.2.7 Physical activity

The model simulates the day between the hours 08:00-21:00, in time cycles of one minute. At each minute (one model cycle), MVPA may be performed by an agent with probability:

$$P_{MVPA,i}(t) = A_i \times P_{MVPA,k}$$

Where $P(t)_{MVPA,i}$ is the probability for performing MVPA at time t for agent i; A_i is the tendency of agent i to be active, and $P_{MVPA,k}$ is the probability for MVPA associated with land use type k, as specified in figure 3. The accumulation of minutes of MVPA during the day is measured for each agent.

Walking is also regarded as an activity, and while walking the agent is assigned a probability for MVPA as specified in Figure 3. The probability for MVPA during school time is divided into different parts of the school day: lessons, recess and PE as specified in Figure 3.

When meeting with friends and during the school day we assume that PA is influenced by friends. Therefore, the tendency of the agent to be active is partially adjusted to the tendencies of his friends:

$$A_i = (1 - \delta) \times A_i + \delta \times \overline{A_f}$$

Where δ is a proportion of the agent's tendency that is adjusted, and $\overline{A_f}$ is the average tendency of the agent's friends.

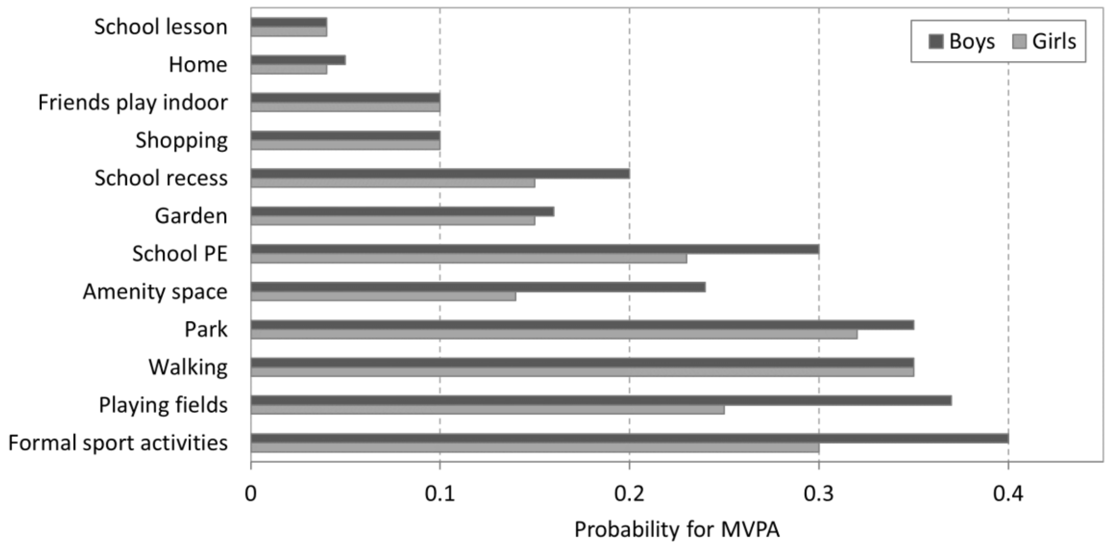

Figure 3: Probability of agents to preform MVPA at each time t when the activity takes place on a specific land use type, for boys and girls. Probabilty estimates are based on analysis of 109 children residing in four large cities in Scotland; Data from SPACES research project (McCrorie et al., 2019).

4.2.8 Travel mode choice

We use empirical data from 713 Scottish children who reported their mode of travel to school for a period of a week-two weeks (Macdonald et al., 2019). Using an ordinal logistic regression, we estimated the daily probability of active travel to school based on distance to school and street walkability (see regression results in: https://github.com/Jonatanalma/ABM_Children_activity). In model initialisation, the ordinal logistic regression is implemented to assigned for each agent the probability of active travel to school given agent's distance to school and street walkability in the area. When travelling to destinations other than school for a distance longer than 300 meters,

agents calculate a walking probability P_{walk} based on distance to destination (Dis), walkability score (Walk) and number of cars (No.Car) in the household, as follows:

$$P_{walk} = 50\% \times +0.7^{(Dis/300)-1} + 30\% \times 0.9^{Walk-1} + 20\% \times 0.7^{No.Car}$$

4.3 Model procedure

The overall daily model's procedure is presented in Figure 4. Each agent follows a daily schedule of activities at different parts of the day. Agents start the day at home. School activities are attended daily by all agents. Formal sport activities schedule and sites are assigned to agents at model initialisation. Meeting with friends is coordinated between agents when school ends. The rest of activities (outdoor play and shopping) are triggered with probabilistic procedures. Before travelling between activities agents select the mode of travel.

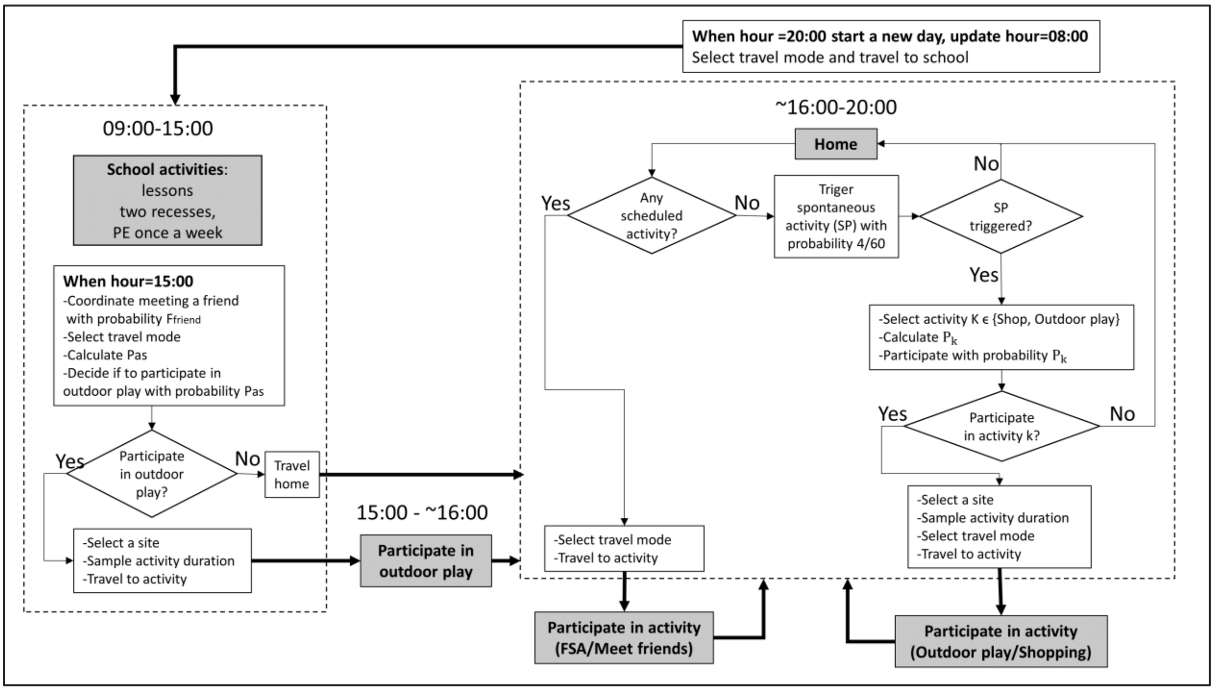

Figure 4: Model's daily procedures. Dashed line signify the two main domains: Home and School. Gray squares denote engagement with an activity where MVPA can be perfomed. Thick arrows denote travel between activities; MVPA can be performed by the agent in case of active travel.

References

Cleland, V., Timperio, A., Salmon, J., Hume, C., Baur, L. A., & Crawford, D. (2010). Predictors of time spent outdoors among children: 5-year longitudinal findings. Journal of epidemiology & community health, 64(5), 400-406.

Dunton, G. F., Almanza, E., Jerrett, M., Wolch, J., & Pentz, M. A. (2014). Neighborhood park use by children: use of accelerometry and global positioning systems. American journal of preventive medicine, 46(2), 136-142.

Evenson, K. R., Catellier, D. J., Gill, K., Ondrak, K. S., & McMurray, R. G. (2008). Calibration of two objective measures of physical activity for children. Journal of sports sciences, 26(14), 1557-1565.

Floyd, M. F., Bocarro, J. N., Smith, W. R., Baran, P. K., Moore, R. C., Cosco, N. G., ... & Fang, K. (2011). Park-based physical activity among children and adolescents. American journal of preventive medicine, 41(3), 258-265.

Hagger, M. S., Chatzisarantis, N., & Biddle, S. J. (2001). The influence of self-efficacy and past behaviour on the physical activity intentions of young people. Journal of sports sciences, 19(9), 711-725.

Koppen, G., Sang, Å. O., & Tveit, M. S. (2014). Managing the potential for outdoor recreation: Adequate mapping and measuring of accessibility to urban recreational landscapes. Urban forestry & urban greening, 13(1), 71-83.

Macdonald, L., McCrorie, P., Nicholls, N., & Olsen, J. R. (2019). Active commute to school: does distance from school or walkability of the home neighbourhood matter? A national cross-sectional study of children aged 10–11 years, Scotland, UK. BMJ open, 9(12).

Macdonald-Wallis, K., Jago, R., & Sterne, J. A. (2012). Social network analysis of childhood and youth physical activity: a systematic review. American journal of preventive medicine, 43(6), 636-642.

McCrorie, P., Mitchell, R., & Ellaway, A. (2018). Comparison of two methods to assess physical activity prevalence in children: an observational study using a nationally representative sample of Scottish children aged 10–11 years. BMJ open, 8(1), e018369.

Marquet, O., Hipp, J. A., Alberico, C., Huang, J. H., Fry, D., Mazak, E., ... & Floyd, M. F. (2019). Short-term associations between objective crime, park-use, and park-based physical activity in low-income neighborhoods. Preventive medicine, 126, 105735.

Maturo, C. C., & Cunningham, S. A. (2013). Influence of friends on children’s physical activity: a review. American Journal of Public Health, 103(7), e23-e38.

Oliver, M., Mavoa, S., Badland, H., Parker, K., Donovan, P., Kearns, R. A., ... & Witten, K. (2015). Associations between the neighbourhood built environment and out of school physical activity and active travel: an examination from the Kids in the City study. Health & Place, 36, 57-64.

Pedroni, C., Dujeu, M., Moreau, N., Lebacq, T., Méroc, E., Godin, I., & Castetbon, K. (2019). Environmental correlates of physical activity among children 10 to 13 years old in Wallonia (Belgium). BMC public health, 19(1), 1-12.

Prince, S. A., Butler, G. P., Rao, D. P., & Thompson, W. (2019). Evidence synthesis: Where are children and adults physically active and sedentary?–a rapid review of location-based studies. Health promotion and chronic disease prevention in Canada: research, policy and practice, 39(3), 67.

Rees-Punia, E., Hathaway, E. D., & Gay, J. L. (2018). Crime, perceived safety, and physical activity: A meta-analysis. Preventive medicine, 111, 307-313.

Remmers, T., Broeren, S. M., Renders, C. M., Hirasing, R. A., van Grieken, A., & Raat, H. (2014). A longitudinal study of children's outside play using family environment and perceived physical environment as predictors. International Journal of Behavioral Nutrition and Physical Activity, 11(1), 1-9.

Rhodes, R. E., McEwan, D., & Rebar, A. L. (2019). Theories of physical activity behaviour change: A history and synthesis of approaches. Psychology of Sport and Exercise, 42, 100-109.

Sallis, J. F., Cervero, R. B., Ascher, W., Henderson, K. A., Kraft, M. K., & Kerr, J. (2006). An ecological approach to creating active living communities. Annu. Rev. Public Health, 27, 297-322.

Scottish-Government. Scottish Index of Multiple Deprivation 2020.gov.scot; 2020. Available from: https://www.gov.scot/collections/scottish-index-of-multiple-deprivation-2020/

Stearns, J. A., Godley, J., Veugelers, P. J., Ekwaru, J. P., Bastian, K., Wu, B., & Spence, J. C. (2019). Associations of friendship and children's physical activity during and outside of school: a social network study. SSM-population health, 7, 100308.

Van Hecke, L., Deforche, B., Van Dyck, D., De Bourdeaudhuij, I., Veitch, J., & Van Cauwenberg, J. (2016). Social and physical environmental factors influencing adolescents' physical activity in urban public open spaces: A qualitative study using walk-along interviews. PloS one, 11(5), e0155686.

Williams, G. C., Borghese, M. M., & Janssen, I. (2018). Neighborhood walkability and objectively measured active transportation among 10–13 year olds. Journal of Transport & Health, 8, 202-209.
