# Supplementary material for: How can an agent-based model explore the impact of interventions on children's physical activity in an urban environment?
Source: Health Place. 2021 Nov;72:102688. doi: 10.1016/j.healthplace.2021.102688 (PMC8633766; doi:10.1016/j.healthplace.2021.102688)
Supplement: Multimedia component 2 — Sensitivity analysis and additional graphs and tables. [file mmc2.pdf]

**How can an agent-based model explore the impact of interventions on children's physical activity in an urban environment?**

Jonatan Almagor, Anne Martin, Paul McCrorie, Rich Mitchell

**Supplementary material**

## 1. Socio-demographic characteristics of the sample from SPACES research project

Using a sample of 109 children we calculated, for different types of land use and sites, the proportion of time that MVPA was measured from the total time spent at the site. Those estimates were used in the ABM as probabilities to perform MVPA based on the agent location. Table 1 presents the socio-demographic characteristics of the sample.

**Table 1:** Socio-demographic characteristics of the sample of children from SPACES research project.

| City | Total | Female | Male | SIMD1 | SIMD2 | SIMD3 | SIMD4 | SIMD5 |
|---|---|---|---|---|---|---|---|---|
| Aberdeen | 13 (12%) | 10 (9%) | 3 (3%) | 1 (1%) | 2 (2%) | 3 (3%) | 0 (0%) | 7 (6%) |
| Dundee | 8 (8%) | 4 (4%) | 4 (4%) | 3 (3%) | 1 (1%) | 0 (0%) | 3 (3%) | 2 (2%) |
| Edinburgh | 55 (50%) | 28 (26%) | 27 (25%) | 4 (4%) | 1 (1%) | 1 (1%) | 6 (6%) | 43 (39%) |
| Glasgow | 33 (30%) | 16 (15%) | 17 (16%) | 7 (6%) | 8 (7%) | 2 (2%) | 6 (6%) | 10 (9%) |
| All cities | 109 (100%) | 58 (53%) | 51 (47%) | 15 (14%) | 12 (11%) | 6 (6%) | 15 (14%) | 62 (57%) |

SIMD- Scottish Index of Multiple Deprivation. SIMD1-lowest quintile, SIMD5-highest quintile.

## 2. Variation in daily MVPA over 25 days

Daily MVPA minutes vary between days because of the different activities the agents are involved in each day. Figure 1a presents the trajectory of MVPA over 25 days for 20 agents simulated in the baseline scenario. The extent of daily MVPA variation over 25 days for the entire agent population is presented in Figure 1b. For about half of the agents the MVPA SD over 25 days comprises 25% or less of their average MVPA. For a third of the agents MVPA SD over 25 days comprises 30% of their average and for 18% the SD is 35%-45% of their daily average over 25 days.

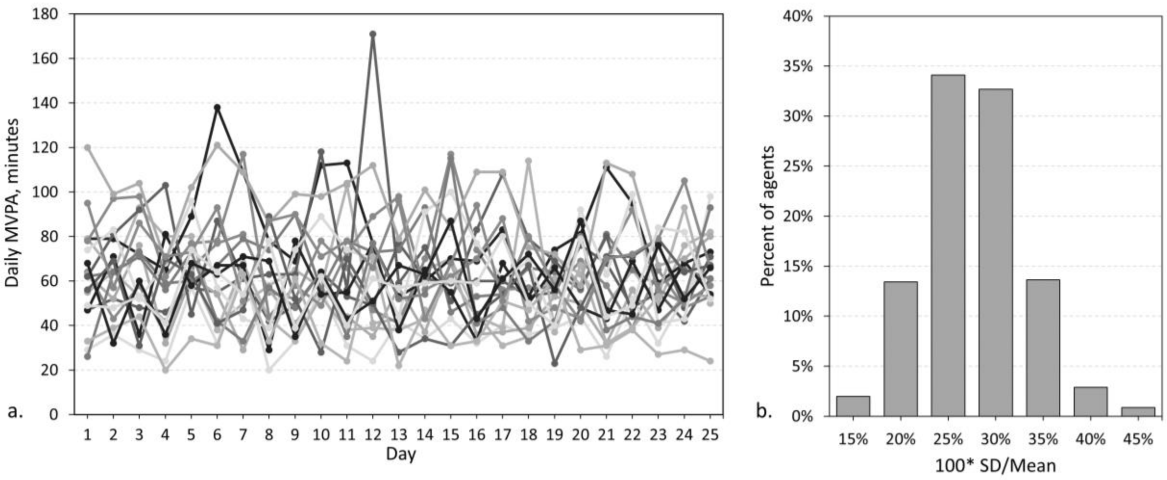


**Figure 1**: Variation of daily MVPA over 25 days. (a) The daily MVPA for a sample of 20 agents simulated in the baseline scenario. (b) Distribution of variation in daily MVPA over 25 days for all

simulated agents for the baseline scenario. The variation in daily MVPA was calculated as the percentage of the SD from the mean for each agent.

## 3. Sensitivity analysis

### 3.1 The impact of travel by car on probability to play outdoors after school (parameter m)

In the ABM we assumed that when the travel mode after school is a car, agents are less likely to engage in outdoor play directly after school, and, therefore, the probability of these agents to engage in outdoor play is multiplied by factor m=1/3 (this reduces the probability by ~67%), (see section 2.4.1 in the manuscript).

In order to test the sensitivity of the ABM to parameter m, we used multiple simulations of the basic scenario where we varied the value of parameter m – the impact on probability to play outdoors after school when the travel mode is a car. We tested increasing values of m in the range of 0%-100%. When m=0% the mode of travel has no impact on outdoor play after school, and as the percentage increases the impact of using the car on the decision to play outdoors increases. When m=100% agents who use a car after school do not engage at all in outdoor play after school.

As can be seen in Figure 2, the variation of m has some impact on MVPA; The m parameter has differential impact on MVPA outcomes for different groups of SEP. Most impact is noticed for the highest SEP (AB). For this group, as the m value increases, the average daily MVPA decreases. The maximal decrease reaches 2 minutes, when m=100%. On the contrary, the daily MVPA of the lowest SEP is not sensitive at all to this parameter and no changes in MVPA are observed. As for C1 and C2 groups, a decrease in daily average MVPA is observed when the m parameter increases, but the decrease is lower compared to AB group.

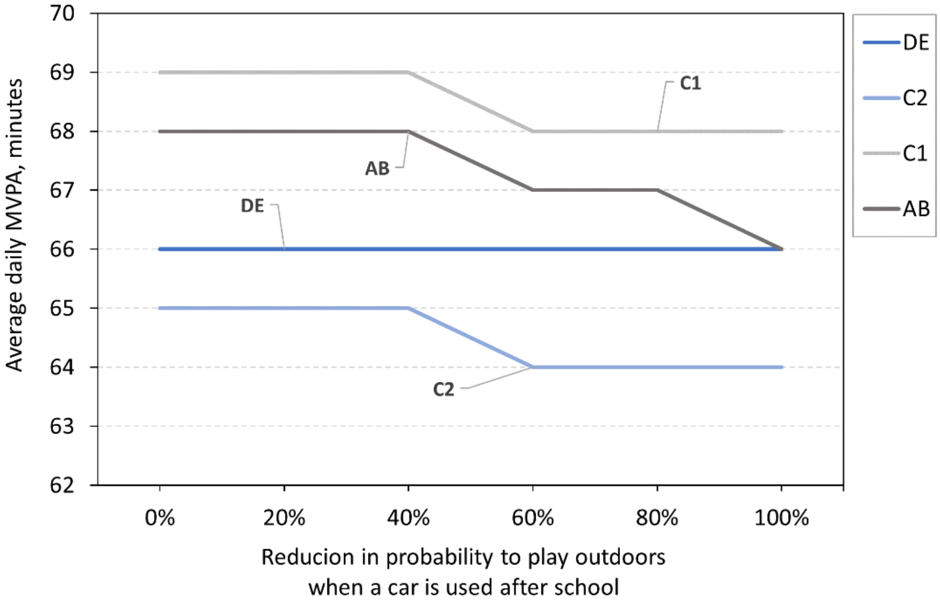


**Figure 2**: Sensitivity analysis for the impact of car usage on outdoor play after school. Line colors represent socio-economic position (SEP). The average daily MVPA (Y-axis) is presented for each SEP for various values of m (X-axis), that is the parameter that impacts the probability to engage in outdoor play after school when the agent's travel mode is a car. The baseline scenario is used in all the simulations.

### 3.2 The impact of the distribution of tendency to be active (*A*) and preference to play outdoors (*o*)

In the ABM initialization, agents are assigned values for tendency to be active (*A*) and preference to play outdoors (*O*). These parameters are randomly drawn from a normal distribution with mean=1 and an assumed standard deviation, to create heterogeneity in the behavior of the agents.

Since we do not know the distribution of these parameters in the population, we assumed some variance that is reflected by the standard deviation of these parameters in the population. In the ABM presented in the manuscript we assumed SD=0.3 for both *O* and *A*. Here we tested the sensitivity of the model output (distribution of daily MVPA) to variation in the SD of these parameters. We tested SD in the range of [0-0.5] for both parameters and simulated 25 combinations of the distribution of O and A in the population using these SD.

Changing the SD of *O* and *A* in the agent population does not affect the daily average of MVPA, but it does influence the shape of daily MVPA distribution in the population. Figure 3 presents the outputs of the sensitivity analysis that reflects the shape of the distribution of daily MVPA in the population using two indicators: 1) the standard deviation of MVPA (Figure 3a) and 2) the percentage of agents with daily MVPA<60 min. As presented in Figure 3, varying the SD of *A* has an impact on the distribution of daily MVPA in the population, while varying the SD of *O* has a minor impact. As the SD of *A* increases from 0 to 0.5, the SD of daily MVPA increases too, from 10 to 19 minutes (Figure 3a) and the percentage of agents with MVPA<60 increases from 29% to 39% (Figure 3b). Increasing the SD of *O* has a minor impact on SD of daily MVPA and the percentage of agents with MVPA<60. Figure 4 presents the distributions of daily MVPA for selected simulations. Note how the distribution of MVPA in the case of no variance in *O* and *A* (Figure 4a) is becoming more dispersed when SD is included to the distribution of parameters *O* and *A* (Figure 4b-f). The stronger impact of parameter *A* on the distribution of MVPA is also apparent; when SD is included to parameter *A* only, the daily MVPA distribution becomes more dispersed (Figure 4c and 4e), while when SD is introduced only to *O*, a minor change is observed in the shape of the distribution (Figure 4b and 4d).

Given that an unknown variation exists in the real-world population in behaviors related to tendency to be active (*A*) and preference to be outdoors (*O*), the estimate of the ABM has uncertainty for the percentage of agents that meets the recommendation of at least 60 minutes of daily MVPA and the decrease in this percentage following the interventions. With that, according to our sensitivity analysis this range is limited to a few percentage points.

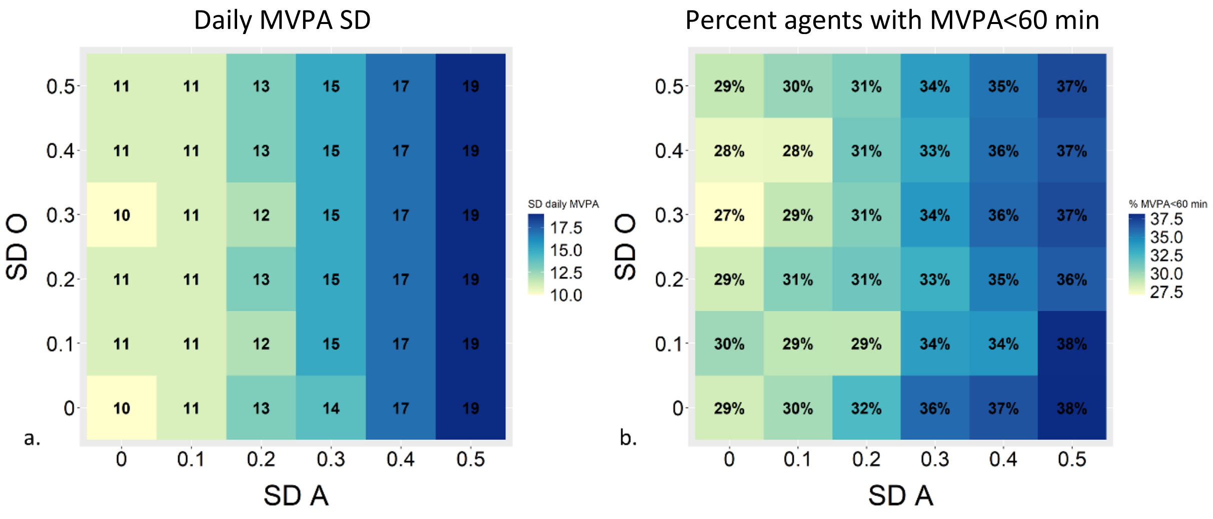

**Figure 3:** The impact of the distribution of parameters *O* and *A* in the agent population on daily MVPA. (a) Daily MVPA standard deviation (SD) of the agent population as dependent on the SD of *O* and *A*. (b) The percentage of agents with daily MVPA<60 minutes as dependent on the SD of *O* and *A*. The graphs were plotted based on results of 36 simulations of the baseline scenario with different combination of the SD of parameters *O* and *A* that were assigned to agents at model initialization.

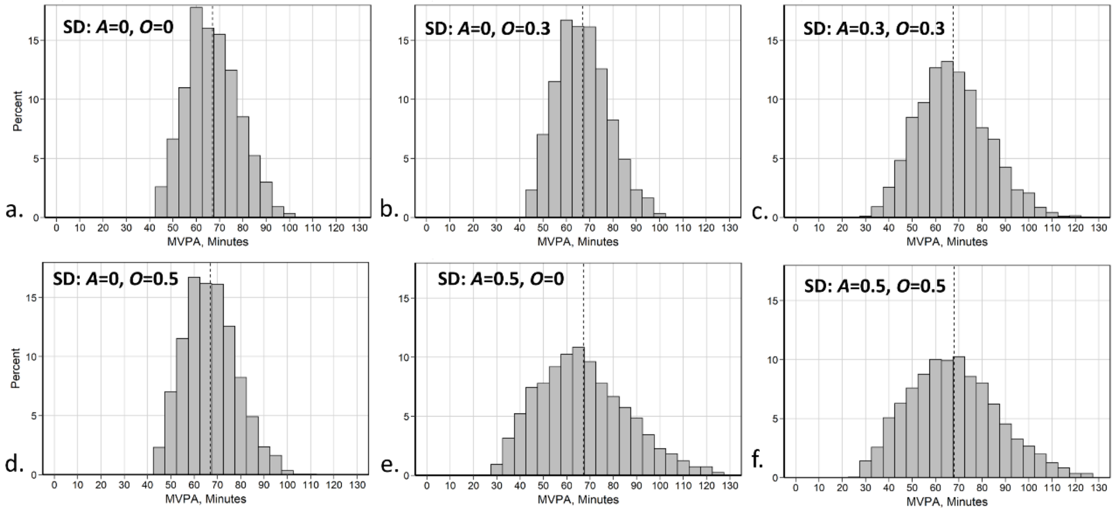


**Figure 4:** Distribution of daily MVPA of the agent population for different distributions of parameters *O* and *A***.** Panels (a)-(f) present simulations that differ by the value of the standard deviation (SD) of the parameters O and A in the agent population. The values of the SD are: (a) *A*=0, *O*=0 (b) *A*=0, *O*=0.3 (c) *A*=0.3, *O*=0.3 (d) *A*=0, *O*=0.5 (e) *A*=0.5, *O*=0 (f) *A*=0.5, *O*=0.5.
